# Local Drug Delivery Strategies towards Wound Healing

**DOI:** 10.3390/pharmaceutics15020634

**Published:** 2023-02-13

**Authors:** Ruchi Tiwari, Kamla Pathak

**Affiliations:** 1 Pranveer Singh Institute of Technology (Pharmacy), Kanpur 208020, Uttar Pradesh, India; 2 Faculty of Pharmacy, Uttar Pradesh University of Medical Sciences, Etawah 206130, Uttar Pradesh, India

**Keywords:** wound, physiology of wound healing, strategies towards wound healing, local delivery systems

## Abstract

A particular biological process known as wound healing is connected to the overall phenomena of growth and tissue regeneration. Several cellular and matrix elements work together to restore the integrity of injured tissue. The goal of the present review paper focused on the physiology of wound healing, medications used to treat wound healing, and local drug delivery systems for possible skin wound therapy. The capacity of the skin to heal a wound is the result of a highly intricate process that involves several different processes, such as vascular response, blood coagulation, fibrin network creation, re-epithelialisation, collagen maturation, and connective tissue remodelling. Wound healing may be controlled with topical antiseptics, topical antibiotics, herbal remedies, and cellular initiators. In order to effectively eradicate infections and shorten the healing process, contemporary antimicrobial treatments that include antibiotics or antiseptics must be investigated. A variety of delivery systems were described, including innovative delivery systems, hydrogels, microspheres, gold and silver nanoparticles, vesicles, emulsifying systems, nanofibres, artificial dressings, three-dimensional printed skin replacements, dendrimers and carbon nanotubes. It may be inferred that enhanced local delivery methods might be used to provide wound healing agents for faster healing of skin wounds.

## 1. Introduction

One of the most crucial characteristics of simple bacteria to complex multicellular creatures is an undamaged outer coating. The largest organ in the human body, the skin serves a variety of purposes. Skin is susceptible to a range of external variables because of its exposure to the environment, which can lead to various skin injuries and damage. A series of physiological events can heal cuts or injuries because skin has remarkable regenerating abilities. The epidermis of the skin is central to several vital organs, including apocrine glands, eccrine sweat glands, and hair follicles with pilosebaceous units [1]. A wound is the result of the “disruption of normal anatomic structure and function,” claims the Wound Healing Society. Depending on how long a wound takes to heal, attention might be given to the acute and chronic kinds. Acute wounds are typically treated effectively with a good possibility of success within a few weeks and are primarily caused by mechanical trauma or surgical procedures [2]. The location, size, depth, and type of an acute wound all affect its nature. The chain of events necessary for wound healing can be disrupted by a variety of disease processes, leading to chronic, non-healing wounds that cause the patient great suffering and need a tremendous number of resources from the medical system. The coagulation cascade, inflammatory pathways, and the cellular components of the immune system are all activated during wound healing, which causes a significant modification of all skin compartments [3]. For expediting in vivo wound healing and minimising scar formation, cellular scaffolds containing fibroblasts, keratinocytes, stem/progenitor cells, or reprogrammed cells have shown promising outcomes.

Depending on the type of wound and the patient, several wound treatments are used, but they all often start with cleaning, involve taking antibiotics, and involve choosing the right dressing [4]. The dressing should encourage autolytic debridement and be non-toxic, non-allergenic, non-adherent, and non-toxic. For deep wounds or after surgery for patients with implanted medical devices, effective local administration of antibacterial chemicals is crucial. Surface-adherent cells may release growth factors that may then affect the timing and result of the creation of a scar as skin cells move into a fibrin-rich temporary matrix to form a scar. A polymer applied therapeutically to the local site of interest may help such local wound-healing activities [5].

When serious cuts are treated with typical wound care techniques, permanent scars are created. As a result, considerable effort has gone into creating alternative therapies that bring back the natural skin’s capacity for regeneration. Cell treatment, growth factor delivery, gene delivery, and other techniques have all been utilised to speed up the healing of non-healing wounds. Drugs and genes can have their half-lives extended, their bioavailability increased, their pharmacokinetics optimised, and their dose frequency decreased using drug delivery systems at the nano, micro, and macroscales [6]. If just extracellular delivery is necessary, microparticle-mediated distribution would have a more long-lasting therapeutic impact since the release kinetics would be slowed by the reduced surface-to-volume ratio. Protein and nucleic acid treatments, on the other hand, would require nanoparticle-mediated transport in order to reach the intracellular targets. Nanotechnology has a fantastic chance to improve currently used medical therapies, standard care, and wound management [7]. It has been discovered that nano drug delivery methods are non-toxic, completely compatible with skin, and favourably generate a helpful moist environment for activating and accelerating the wound healing process. 

When comparing different drug delivery methods, tissue-engineered scaffolds are particularly pertinent to wound healing since they can act as a depot for medicines [8]. Additionally, they can serve as wound dressings to physically protect the wounds. Therefore, drug-incorporated scaffolds hold great promise for speeding the healing of chronic wounds through a combination of mechanisms. By acting through immune regulation, paracrine actions, and differentiation into epidermal and dermal cells to restore the injured skin, stem cell-based treatments show increasing promise [9]. The application of particular stem cells has emerged as a promising strategy to overcome the drawbacks of conventional treatments, offering the potential to improve the healing of chronic wounds as well as speed up the process of wound healing for acute wounds [10]. There are several different antibiotic formulations that can be used topically on wound sites. Medicine delivery devices are particularly crucial because when a drug is given systemically, the poor vasculature in the wound bed can prevent the drug from effectively reaching the healing tissue. Additionally, complicated drug delivery systems that can transport the active ingredients in the right quantity to the right area are needed due to the side effects of some medications, the short half-lives of biological components, and the dynamic nature of the wound environment [11].

The physiology of wound healing as well as current advancements in wound healing techniques, with a focus on local medication delivery elements, are critically examined in this review article.

## 2. Physiology of Wound Healing Process

Skin wound healing is an intriguing biological process that has served mammals well throughout evolution. Skin wound healing is a crucial phase for survival that culminates in wound closure because of its essential roles as a physical, chemical, and bacterial barrier. The process of cellular, humoral, and molecular mechanisms involved in skin wound healing is dynamic, tightly regulated, and can take years to complete [12]. The epithelialisation of skin wounds depends on the details of the lesion, including its location, depth, size, microbial contamination, patient-related medical problems, genetics, and epigenetics. Following a skin injury, the exposed sub-endothelium, collagen, and tissue factor will stimulate platelet aggregation, which in turn causes degranulation and releases chemotactic factors (chemokines) and growth factors to form the clot [12]. By following all of the aforementioned steps, successful haemostasis will be achieved. 

**Figure 1:**
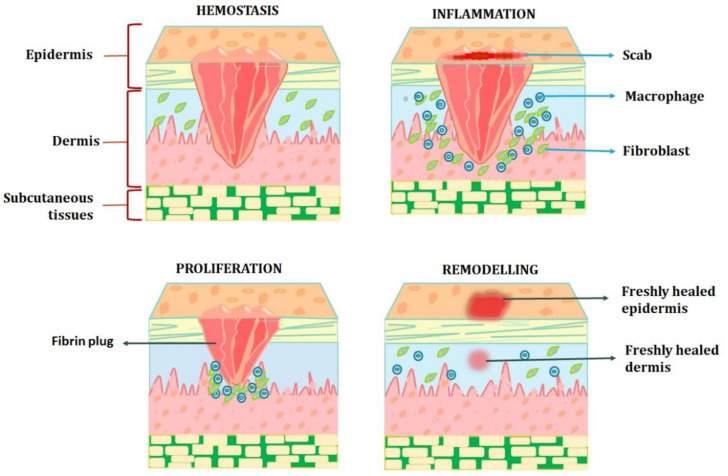
Physiology of wound healing. Haemostasis: The immediate response to a surgical injury is the vasoconstriction of blood vessels at the point of injury. Inflammatory process: brings nutrients to the area of surgery, removes debris and bacteria, and provides stimuli for wound repair. Proliferation: involves processes of angiogenesis, granulation tissue production, collagen deposition, and epithelialisation. Remodelling: Type 1 collagen replaces the type 3 collagen found in granulation tissue, and a scar forms.

A skin wound can heal completely through regeneration or repair. Skin repair shows an unspecific kind of healing in which the lesion heals by fibrosis and scar formation, in contrast to regeneration, which depicts the specific substitution of the tissue, such as the superficial epidermis, mucosa, or foetal skin. Unfortunately, the latter is the primary mode of adult skin wound healing [13]. The pathophysiology of chronic wounds is still poorly understood; however, it is known that they remain in the inflammation stage of the healing process rather than progressing further. Critical obstacles to the physiologic healing of chronic wounds include impaired vascularisation and the resulting hypoxia, the inability to move on to the healing phase, extended and exacerbated inflammation, and the incapacity of immune cells to manage bacterial infection. Normal wound healing can be hampered by prolonged wound-healing phases or overly aggressive reactions of the organism to the damage [14]. The majority of chronic wounds heal through fibrosis, which produces an excessive quantity of connective tissue rather than regeneration. Additionally, persistent inflammation is followed by fibrosis, and fibrosis-healing wounds have been discovered to have higher levels of pro-inflammatory mediators (such as TGF-β) (Figure 1). Unnecessary fibroblast proliferation, neovascularisation, and increased collagen and fibronectin synthesis are all results of poorly controlled growth factor activity. Additionally, a wound contracts too much and for too long, causing fibrotic scar tissue to grow. Keloids and hypertrophic scars are two different types of pathological scars that can result from an injury. In this regard, there is active current study on the change from the inflammatory to the proliferative stage of wound repair. Three to five phases that overlap in both time and location can be artificially created to represent the various stages of wound healing [12,13,14,15,16].

### 2.1. Haemostasis and Coagulation: Vascular Mechanism

**Figure 2:**
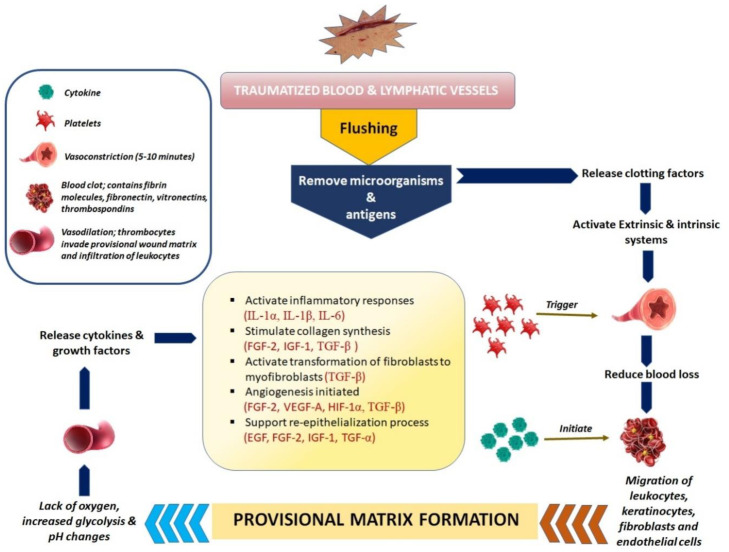
Vascular mechanism for Haemostasis and coagulation. This mechanism is regulated by a dynamic equilibrium between endothelial cells, thrombocytes, coagulation, and fibrinolysis, which also influences the amount of fibrin deposited at the wound site and affects how quickly the reparative processes proceed.

The thrombogenic sub-endothelium is initially exposed to platelets by haemorrhage into the wound. When the skin is wounded, bleeding typically occurs to help remove microorganisms and/or antigens from the wound. The main goal of the vascular mechanism is to stop exsanguination in order to maintain the integrity of the circulatory system and ensure important organs’ abilities to function unharmed despite the injury. The long-term provision of a matrix for the invasive cells that are required in the later stages of healing is the second goal. Bleeding causes haemostasis to be activated, which occurs by exudate components such as clotting factors [17]. Vasoactive chemicals such as serotonin and catecholamines work through particular endothelium receptors to constrict nearby blood vessels. Leukocytes, red blood cells, and plasma proteins can enter the body by way of smaller arteries when they are triggered to vasodilate. A local perfusion failure with subsequent oxygen deprivation, increased glycolysis, and pH alterations is explained by the life-saving vasoconstriction with clot formation. Following vasoconstriction, there is a vasodilation during which thrombocytes infiltrate the temporary wound matrix [18]. Hyperaemia, a localised redness, and wound oedema are additional signs of vasodilation. Platelets are essential to this stage, as well as the overall healing process, because, in addition to establishing early haemostasis, they also release a number of cytokines, hormones, and chemokines that initiate the subsequent stages of healing. In order to prevent further blood loss, the coagulation cascade is activated alongside haemostatic processes through intrinsic and extrinsic routes, causing platelet aggregation and clot formation. The presence of fibrinogen in the exudate triggers the clotting mechanism, which causes the exudates (blood devoid of cells and platelets) to coagulate (Figure 2). This, along with the construction of a fibrin network, results in a clot in the wound, which stops bleeding [19]. The fibrin, fibronectin, vitronectin, and thrombospondin molecules found in the blood clot serve as the provisional matrix, a scaffold for the migration of leukocytes, keratinocytes, fibroblasts, and endothelial cells as well as a source of growth factors. Additionally, this stage is where the inflammatory process starts. This stage is occasionally referred to as the “lag phase” because the organism must coordinate the recruitment of numerous cells and components for the healing process while the wound lacks mechanical strength [18,19,20].

### 2.2. Inflammation: Cellular Mechanism

The inflammatory phase starts practically immediately after haemostasis and lasts for around 3 days, often starting as soon as a few minutes after damage and lasting up to 24 h. Through the production of histamine and serotonin, the release of protein-rich exudate into the wound produces vasodilation, allowing phagocytes to enter the wound and devour dead cells (necrotic tissue). It starts the complement cascade and sets off molecular processes that allow neutrophils, whose primary job it is to fight infection, to infiltrate the wound site. To remove bacteria, foreign objects, and injured tissue, the neutrophils must first perform the vital process of phagocytosis [21]. Within the first 24 h, many neutrophils arrive on the scene as the initial leukocytes. Macrophages quickly follow neutrophils because they are drawn to the consequences of neutrophil death. In the wound, phagocytic cells such as macrophages and other lymphocytes start to sweep away debris and microorganisms. 

**Figure 3:**
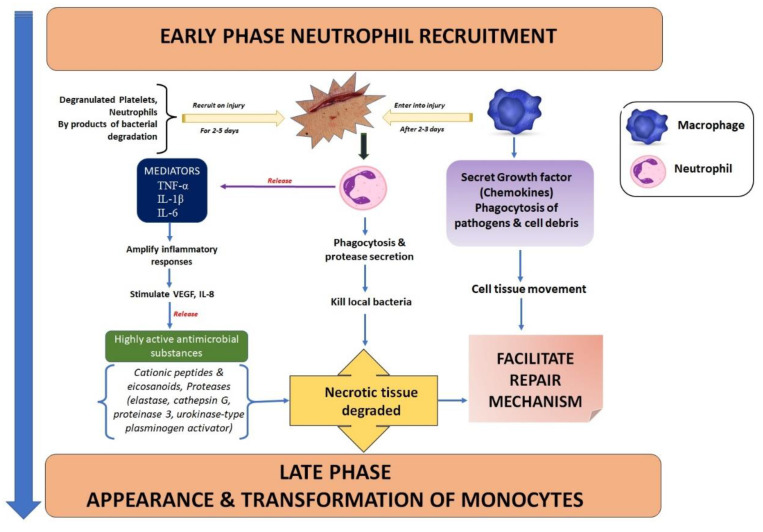
Inflammatory phase following a cutaneous incision. Inflammatory cell invasion: Early inflammatory phase activates the complement cascade and initiates molecular events while in the late phase, macrophages appear in the wound and continue the process of phagocytosis.

About 48 h after the injury, these macrophages infiltrate and remain there until the inflammatory phase is over. Long considered the main cell in the healing of wounds, macrophages appear to coordinate the most crucial stages of healing (Figure 3) [22]. Recent studies have looked at their role in both poor healing and scarring, despite the fact that they are essential to normal recovery. Re-epithelialisation, granulation tissue development, angiogenesis, wound cytokine production, and wound contracture are intricate processes involving macrophages. The succeeding procedures depend on phagocytotic activity because acute wounds with a bacterial imbalance are unable to heal. Inflammation is brought on as circulating monocytes enter the tissue and quickly mature into adult macrophages. By activating type-1 macrophages (M1), phagocytosis eliminates pathogens, foreign objects, necrotic neutrophils, and wound dermis from the diseased area. They generate a number of cytokines and proinflammatory mediators. Mastocytes are sensitive to tissue damage and play a crucial role in the healing process by secreting a number of cytokines that promote the recruitment of white blood cells [17,18,19,20,21,22]. T cells enter the wound site and control a variety of processes. Monocytes, cytokines, macrophages, corneocytes, big granular lymphocytes, T-lymphocytes, basophils, granulocytes, and vascular endothelial cells are all involved in wound regeneration. IL-1, TNF-α, IL-6, VPF, TGF- β, and IGF-1 are among the cytokines produced by monocytes, which develop into macrophages. Along with corneocytes, fibroblasts, granulocytes, and vascular endothelial cells, neutrophils, such as T lymphocytes and basophils, are important makers of TNF-α, IL-10, and other chemokines. VEGF, IGF-1, and TGF- β are all produced by macrophages [12,13,14]. As a result, each of the cells mentioned above participates in the intricate process of tissue repair. Subsequent repair mechanisms of an adult depend on cell and tissue movements that are caused by growth factor and cytokine signals, which are provided by the inflammatory response to injury. Epithelial cells and fibroblasts travel to the injured location during the migration phase to replace lost and damaged tissue. These cells quickly spread over the wound beneath the dried scab (clot), regenerating from the borders and thickening the epithelium [23].

### 2.3. Proliferation

Because the proliferative stage of wound healing is highly metabolic with an increased demand for oxygen and nutrients, the restoration of blood flow is essential. For phagocytes undergoing a respiratory burst to effectively combat pathogens, the presence of oxygen in cutaneous wounds is also essential.

#### 2.3.1. Epithelialisation

The multiplication and inflow of keratinocytes close to the wound’s leading edge indicate epithelialisation. Re-epithelialisation begins during the proliferative phase of wound healing, roughly 16–24 h after injury, and continues through the second and third phases. It is characterised by fibroblast migration and the deposit of a recently created extracellular matrix, which takes the place of the temporary fibrin and fibronectin network [18]. Capillary budding and the synthesis of the extracellular matrix are also involved in the repair phase to fill in the gaps left by the debridement of the wound. Hair follicle and apocrine gland bulbs contain stem cells that start to develop into keratinocytes, repopulate the stratum basale, and move over the edge of the wound. They connect close to the inner edge of the wound once they come into contact with the mesenchyme of the extracellular matrix (ECM), and they start to lay down a new basement membrane. Keratinocytes are crucial for maintaining the barrier as well as for its repair after injury through a process called epithelialisation. Undifferentiated keratinocytes convert into differentiated non-dividing cells during differentiation as they move upward to eventually give rise to the cornified envelope [24]. The process of differentiation is mediated by three main mitogen-activated protein kinase (MAPK) pathways, which are activated by a variety of stimuli, including calcium influx, epidermal growth factor (EGF), and TNF-α. K6, K16, and K17 keratins are upregulated in migrating keratinocytes, which is thought to augment the viscoelastic characteristics of moving cells. In addition to producing paracrine and autocrine signals that are directed at surrounding keratinocytes, activated keratinocytes also alert fibroblasts, endothelial cells, melanocytes, and lymphocytes. In order to coordinate the activity of neighbouring cell types in the repair of damaged tissue, these responses are crucial [25].

#### 2.3.2. Angiogenesis

In response to tissue injury, the complicated mechanism of angiogenesis is heavily controlled by signals from the ECM and serum. Activated macrophages, the epidermis, and soft tissue wounds can all produce angiogenesis. Gelatinase A is released by endothelial cells exposed to thrombin, and it aids in the local disintegration of the basement membrane, an essential first step in angiogenesis. Many soluble factors, most notably VEGF-A, positively influence the initiation of angiogenesis. Due to its powerful angiogenesis and vasopermeability activity, VEGF, a growth factor belonging to the PDGF family, was initially called the vasopermeability factor [22]. The VEGF family in mammals consists of five members (VEGF-A, -B, -C, and -D and placenta growth factor). In the wound, thrombin increases cellular receptors for VEGF. Due to a hypoxic gradient between injured and healthy tissue, expression of the HIF-1 gene causes the synthesis of VEGF. Nitric oxide generation by endothelial cells is also influenced by hypoxia [25]. To increase local blood flow, nitric oxide encourages angiogenesis and vasodilation. The most important proangiogenic factor in wound healing is VEGF-A, which is produced in response to hypoxia. A powerful proangiogenic mediator, VEGF-A also raises vascular permeability, which adds to wound oedema. Other factors, such as cardiac ankyrin repeat protein, as well as VEGF-A, FGF-2, PDGF, TGF-β family members, and other factors also encourage wound angiogenesis. Numerous highly technical depletion investigations on skin have shown that macrophages are a significant contributor to overall proangiogenic stimulation. Thus, it appears that in the healing wound, inflammation and the subsequent angiogenic response are related [26].

#### 2.3.3. Granulation Tissue Formation

Granulation tissue normally grows from the wound’s base and can cover any size wound. Chronic wound formation can be caused by any mistakes in the granulation tissue creation process. Fibroblasts, freshly sprouting blood vessels, and immature collagen make up granulation tissue (collagen type III) [25]. In this stage, some fibroblasts will also start differentiating into myofibroblasts, which can contract to close wound edges that are protruding from the body. TGF-β and PDGF, which are generated by inflammatory cells and platelets, entice fibroblasts and myofibroblasts from the surrounding tissue to move into the wound [27,28]. Through the creation of granulation tissue brought on by hypoxia, increased lactate, and different growth factors, healing through secondary intention is accomplished. Epithelisation over this granulation is a necessary step in healing by secondary intention, followed by substantial remodelling [29]. Any medicine that prevents the growth of new blood vessels may hinder the healing of wounds. In addition to the collagen matrix, fibrinogen, fibronectin, and hyaluronic acid, macrophages, proliferating fibroblasts, and vascularised stroma also make up the acute granulation tissue that takes the place of the fibrin-based provisional matrix. The blood vessels become less dense as collagen builds up, and the granulation tissue gradually reaches maturity to form a scar [30].

### 2.4. Remodelling Phase

Through cell apoptosis, the production of granulation tissue is stopped. Cellular connective tissue is generated during tissue maturation or remodelling, and the newly formed epithelium is strengthened. From a few months to around two years, cellular granular tissue transforms into an acellular mass. Fibroblasts and macrophages are important players in remodelling. Several growth factors, including TGF-β, PDGF, and FGF, which are stimulated during tissue injury and repair, control remodelling. Although the function of growth factors in the development of scars is not entirely known, TGF-β is assumed to be significant [26]. By boosting the development of tissue inhibitors of metalloproteinase and causing an increase in collagen deposition, this factor is known to decrease pro-collagenase production and enzyme activity. The collagen that was put down during proliferation is eventually replaced by a more stable interwoven type III collagen during remodelling as the water content of the wound decreases. In order to maintain a precise balance between synthesis and degradation and promote normal healing, regulatory mechanisms carefully govern the remodelling of an acute wound. Concurrent with the development of the granulation tissue, the extracellular matrix begins to be synthesised during the proliferative and remodelling phases. The proliferative phase’s collagen III is now being replaced by the more robust collagen I. Later, the myofibroblasts generate wound contractions through various collagen attachments and aid in reducing the surface area of the scar [31,32].

## 3. Wound Healing Strategies

### 3.1. Cellular Activity Initiators

DNA synthesis is promoted in fibroblast cells by secretions from healthy wounds. Conversely, the same fibroblasts are inhibited by the secretions from long-lasting, nonhealing wounds, such as leg ulcers. Interestingly, heating the fluid contents of a chronic wound denatures them, removing the inhibitory impact and restoring fibroblast growth. As before, fibroblasts from chronic wounds have the worst response to growth factors than fibroblasts from acute wounds, suggesting that the fibroblasts in chronic wounds are also harmed. The most promising biomarkers are proteases and cytokines. Traditional medicines such as honey, curcumin, and tannin have been studied using modern pharmaceutical practices to learn how they affect cellular activity. One would anticipate that cytokine release, which represents neutrophil and macrophage activity, would increase in an effort to trigger a fibroblast response if fibroblasts stop responding. The pro-inflammatory cytokines IL-1 and TNF-α were found in higher concentrations in non-healing wounds than in healing wounds. When the healing starts, the levels significantly decrease. An electromechanical coupling bio-nanogenerator made of extremely discrete piezoelectric fibres was created by Tong et al. [33]. By using the inherent force of the cell, it can produce a surface piezopotential up to millivolts, providing in situ electrical stimulation for the living cells. Additionally, the three dimensional structure of bionanogenerator encourages growth of ECM. Bio-nanogenerators successfully support cell viability and development as a result, but more crucially, they maintain the cell’s unique functional expression. 

Several methods were used to modulate macrophages, including blocking IL-1 or TNF-α, inhibiting the inflammasome pharmacologically, neutralising MCP-1, and chelating iron with desferrioxamine. Sulphated hyaluronic acid is internalised by macrophages after being identified by CD44 and the scavenger receptors CD36 and LOX-1. Most notably, it prevents the phosphorylation of the transcription factors including pNFkB, pSTAT1, and IRF5 that are involved in M1-like activation states and the production of pro-inflammatory genes. Sulphated hyaluronic acid regulates macrophage activation in vivo. Inhibiting the secretion of growth factors by macrophages, blunting the immune system’s response to presented antigens, blocking the conversion of membrane phospholipids to arachidonic acid, and reducing vascular permeability are a few of the ways that steroids modify the inflammatory process at various stages of the cascade of wound healing.

By altering fibroblast activity and proliferation, anti-fibrotic medications such as mitomycin C and 5-FU stop the formation of scars. By disrupting pyrimidine metabolism, the anti-proliferative activity of 5-FU is mediated. By preventing the production of thymidine nucleotides, it prevents DNA synthesis, leading to cell death. It has long-lasting effects on Tenon’s fibroblasts and can effectively limit fibroblast development [34]. By reducing to an alkylating agent, mitomycin C is activated and subsequently works by cross-linking DNA. Mitomycin C can impede not just DNA replication but also mitosis and protein synthesis. Hypermongone C, a polycyclic polyprenylated acylphloroglucinol, was shown by Ehsan et al. [35] to have the ability to speed up wound closure by simultaneously boosting fibroblast proliferation and migration, encouraging angiogenesis, and inhibiting pro-inflammatory cytokines. This substance comes from the Hypericum plant family, which has long been used to cure wounds. There are now more opportunities for the combination of therapeutic strategies in multifunctional ECM-based wound dressings thanks to ECM-based materials that have already been investigated for the delivery of antimicrobials and sustained release of angiogenic and pro-fibrotic growth factors in wound healing. Proteomic and microbiome analyses, gene sequencing, high-resolution imaging, and single-cell laser capture are some of the new technologies that may offer comprehensive information that helps define macrophage subtypes with distinct activation profiles in physiologically healing wounds and recognise their dysregulation in chronic wounds. Improvements in collagen production and angiogenesis have been reported using microspheres carrying FGF-10 [36].

### 3.2. Collagen Synthesis Activators

In addition to providing resident cells with structural support, collagens, which are found in the dermis as fibrillar proteins, also control resident and inflammatory cell function. Since collagen plays a crucial role in the healing of wounds, chemicals that alter the molecular processes that cause collagen synthesis have been recognised as effective wound-healing medications. In in vitro and in physiological settings, the collagenase from the bacteria *Clostridium histolyticum* hydrolyses triple-helical collagen utilising synthetic peptides as substrates. The anti-collagenase activity of phytoconstituents and crude extracts from natural resources has received extensive study. Numerous phytoconstituents found in plants, including polyphenols with collagenase inhibitory activity, such as flavonoids, terpenoids, glycosides, vitamin E, vitamin C, phenolic acids, and tannins, are abundant. Reduced collagen production and stability, slower re-epithelialisation, and an elevated susceptibility to infection have all been linked to vitamin A insufficiency [37].

*Ascorbic acid* (vitamin C), is crucial for the manufacture of collagen. The collagen that is produced in scurvy is unhydroxylated, relatively unstable, and prone to collagenolysis. A lack of vitamin C causes fibroblasts to create unstable collagen, which offers a flimsy foundation for repair. Although it is generally known that animals with vitamin C deficiencies take longer to repair wounds, it is unclear whether oral vitamin C supplements speed up the healing process. A lack of vitamin K impairs the generation of the clotting factors (factors II, VII, IX, and X), which leads to bleeding disorders, the formation of haematomas, and consequent negative effects on wound healing [38]. In order to transport oxygen, iron is needed. The immune system and other mineral systems depend on substances such as copper and zinc. Lack of zinc causes the production of granulation tissue to be disrupted. *Aloe vera* is a natural substance that is still frequently used and acknowledged in domestic and professional settings as a tool for treating wounds. Aloe vera is primarily recognised for its ability to lessen pain in burn wounds, but it also helps wounds produce more collagen.

Collagenase and dexpanthenol-containing ointment formulations have been used to speed up re-epithelialisation, reduce fibroblast proliferation, and rebuild the ECM. Due to their advantageous effects, topical preparations of growth factors have recently been investigated in wound healing. Growth factors have a low bioavailability, which limits their use because they are quickly removed from the wound site. Emerging strategies that topically apply growth factors are nanoparticle-encapsulated and have improved stability and bioavailability to the wound area aim to address this limitation. Rats with parenchymal lung lesions experienced better wound healing and an increase in the presence of immature collagen after intraperitoneal glutamine treatment [39]. Dietary glutamine supplementation increased the collagen density in colonic anastomoses in rats, indicating that fibroblasts can synthesise enough glycine for collagen production, whereas they need a source of extracellular glutamine. Glutamine availability can also control collagen mRNA expression in fibroblasts [40].

### 3.3. Angiogenesis Activators

By acting as a chemoattractant for neutrophils, macrophages, and fibroblasts, TGF-β promotes the development of granulation tissue. As a result, TGF-β is a crucial regulator of angiogenesis during the healing of wounds because it controls cell division, migration, capillary tube formation, and ECM deposition. Through the production of particular proteins, gene augmentation brings about the return of normal cellular function. By delivering DNA or mRNA into the target cells, one can enhance genes. Exciting new choices for treating chronic wounds will be made possible in the following ten years by wound dressings that contain sustained nucleic acid delivery systems for promoting angiogenesis and therapy that targets the underlying morbidities. Reactive oxygen species (ROS)-scavenging hydrogel and oxygen-release microspheres were combined to create a sustained oxygenation system by Ya et al. [41]. In diabetic wounds, the continuous release of oxygen increased keratinocyte and dermal fibroblast survival and migration, encouraged the development of angiogenic growth factors and angiogenesis, and reduced the expression of pro-inflammatory cytokines. The pace of wound closure was greatly accelerated by these effects. With its interactions with a number of immune and non-immune cells, C1q is a well-known starter of the complement classical route and induces complement activation-independent activities. One of the probable targets of C1q, which binds to receptors found on cell surfaces and promotes inflammation, are endothelial cells. C1q has a special and hitherto unknown role in promoting angiogenesis through the globular heads. The ability of C1q to stimulate the growth of new blood vessels in both in vitro and in vivo models of wound healing provided evidence for its angiogenic action [42]. In order to promote the healing of wounds, Jin et al. created temperature-responsive nanobelt fibres that contain vitamin E. A correct matrix elasticity that encourages mesenchymal stem cell adhesion and angiogenesis was given by the cross-linked collagen sheets created by the nanoparticles [43]. Additionally, the scaffold encouraged endothelial cells to form tubes. The therapeutic potential of nanoparticle formulation results in the stimulation of angiogenesis. The flexibility of collagen sheets was improved by praseodymium-cobaltite nanoparticle cross-linking for the pro-angiogenic and stem cell differentiation ability [44].

### 3.4. Cytokine and Growth Factor Activators

Small, secreted proteins called cytokines influence not only the activity of immune cells but also that of other cells. Interleukins, lymphokines, and several related signalling molecules, such as TNF-α, interferons, and others, are among them. Through the activation of cell surface TGF-β serine/threonine type I and type II receptors and the activation of a Smad3-dependent signal, active TGF-β1 induces the fast chemotaxis of neutrophils and monocytes to the wound site. Leukocytes and fibroblasts that have expressed TGF- β1 are then stimulated to produce additional cytokines, such as TNF-α, IL-1β, and PDGF, as well as chemokines, which are all part of a cytokine cascade. Such factors serve to maintain the inflammatory cell response by influencing neutrophil and monocyte recruitment and activation. TGF-β and other cytokines that activate their corresponding cell surface receptors cause intracellular signalling pathways to be activated, which, in turn, causes target cell populations to respond phenotypically and functionally. NF-κB, early-growth response 1 (EGR1), Smads, and MAPK are some of the upstream signalling cascades involved in acute tissue injury. These cascades activate many cognate target genes, including adhesion molecules, coagulation factors, cytokines, and growth factors. The platform upon which circulating leukocyte-expressing counter-adhesion molecules (integrins, selectins, and Ig superfamily members) tether allows them to sense the microenvironment and react to chemotactic signals at the site of tissue injury. This is accomplished by cytokine-induced enhancement of adhesion molecules (VCAM-1, ELAM-1, and ICAM-1) on the endothelium. In response to various chemotactic cues, transmigration from within to outside the artery wall is made possible by interactions between adhesion molecules on blood leukocytes and endothelium. Numerous chemokines are generated in addition to the chemotactic action of TGF-β1 for neutrophils and monocytes to attract leukocytes to the site of tissue injury. Depending on where the cysteine residues are located, several families of related molecules serve as representations of chemokines [45].

Growth factors such as PDGF, TGF-β, and EGF are secreted when platelets degranulate and release alpha granules. PDGF plays a crucial role in luring neutrophils to the wound site to eliminate contaminated germs, coupled with proinflammatory cytokines such as IL-1. A number of pro-inflammatory cytokines (IL-1 and IL-6) and growth factors (FGF, EGF, TGF-β, and PDGF) are released by macrophages to support the development of granulation tissue. One of the first substances to be created in response to skin lesions is a substance known as a proinflammatory cytokine, which controls immune cell actions during epithelialisation. TNF-α, IL-1, IL-6, and IL17 are the main proinflammatory cytokines that play a role in the inflammation phase of wound healing. They also play a role in the epithelialisation phase by promoting cell proliferation and differentiation and mobilising local stem/progenitor cells. Cytokine modulators are a new class of medicinal drugs that prevent fibrogenesis. Scarring and fibrosis of the skin are frequently the results of excessive fibrogenesis [46]. As a different strategy to prevent fibrosis, the modulatory effects of natural compounds such as terpenes and honey should be taken into consideration. Mitomycin P modulators such as buckwheat and acacia honey should be taken into account as substances reducing scarring and encouraging re-epithelisation. Terpenoids are frequently present in essential oils and serve as cytokine suppressors, increasing the production of IL-10 and the anti-inflammatory cytokines TNF- and IL-1 [47].

### 3.5. Antimicrobials

There is a lot of debate about the application of topical antibiotics to wounds. Topical antimicrobial agents are described as substances that can eliminate, suppress, or lessen the number of bacteria. These substances include disinfectants, antiseptics, and antibiotics. Topical antimicrobial medicines are essential to topical burn care because they are used to prevent and control infection. The ideal topical preventive antimicrobial agent would be able to enter necrotic tissue without being absorbed by the body, have a broad spectrum of activity, a lengthy duration of action, have low toxicity, and have several other qualities [48].

A renewed interest in silver-based medications is a result of concerns about bacterial resistance. For thousands of years, silver has been utilised in medicine for its antibacterial properties. A good environment for wound healing can be created by using topical antimicrobials that do not impede epithelial outgrowth and deliver a high concentration of active components to devitalised, devascularised, and, perhaps, necrotic wounds. Topical antibacterial use may reduce the requirement for extensive debridement and subsequent grafting as well as wound deepening. Although microorganisms are present in every wound, the majority do not become infected and heal properly. In these circumstances, the immune system of the host and the bioburden of the wound are in equilibrium [49]. NF-κB is nuclear translocated as a result of toll-like receptor (TLR) stimulation through intracellular signalling from adapter proteins, which, in conjunction with mitogen-activated protein kinases, triggers the transcription of a variety of inflammatory cytokines, chemokines, antimicrobial peptides, and costimulatory factors. According to wound specialists, there is a threshold over which antimicrobial intervention is necessary when bacteria loads are more than or equal to 104 CFU/g. Silver compounds are among the antibacterial agents used in burn treatment. In cases when surgery is either not possible or would not be the first option right away, such as in cases of facial burns, silver sulphadiazine is frequently utilised and acts on burn eschar to restrict the area of non-viable tissue [50,51,52,53,54,55]. Since silver is a natural broad-spectrum antibiotic, there has not been any bacterial resistance to its treatments yet. There are numerous types of silver, including silver oxide, silver nitrate, silver sulphate, silver salt, silver zeolite, silver sulfadiazine, and silver nanoparticles. When silver cations come into touch with liquid, they are freed from their carrier dressings. Depending on the dressing employed, there are significant differences in the pace, duration, and peak level of silver released. Once discharged, silver kills germs in a variety of ways. The healing of both acute wounds and chronic wounds is currently aided by various forms of silver. Antiseptic and antibiotic dressings are the two primary categories into which antimicrobial dressings can be divided.

Due to their good efficacy and tolerability among the various antimicrobial agents available, iodophor-based formulations such as povidone iodine have remained well-liked after decades of usage for antisepsis and wound healing applications. Povidone iodine has been reported as having a wide range of activity, the capacity to penetrate biofilms, a lack of related resistance, anti-inflammatory qualities, low cytotoxicity, and good tolerability. In clinical practice, no adverse effects on wound healing have been noted. Another antimicrobial agent that penetrates burned tissue is cerium nitrate. It has a wide range of activity against Gram-positive and Gram-negative bacteria, as well as fungal species, and is highly effective when used in conjunction with silver sulphadiazine [56]. Povidone iodine is fully hazardous to keratinocytes and fibroblasts at concentrations greater than 0.004 and 0.05%, respectively [57,58,59,60,61,62,63,64,65,66,67,68,69,70,71,72,73,74]. According to in vitro testing, cadexomer iodine is not harmful to fibroblasts at doses up to 0.45%. At doses between 0.2 and 0.001%, chlorhexidine also exhibits dose-dependent toxicity to fibroblasts. Antiseptics are primarily used to avoid infection, reinfection, and probable disruption of wound healing. Antiseptic therapy also has the secondary purpose of promoting wound healing by stimulating cell growth and regeneration. These effects have been shown for polyhexamethylene biguanide, in addition to pure microbicidal action (polyhexanide). Antiseptics also have additional beneficial benefits, such as wound cleaning, which can aid in debridement. Effective antiseptics for local wounds include polyhexanide and octenidine dihydrochloride. Nitrofuran and natrium fusidate are other antibacterial substances (Table 1) [48,49,50,51,52,53,54,55,56,57,58,59,60,61,62,63,64,65,66,67,68,69,70,71,72,73,74,75,76,77,78,79,80,81,82,83,84,85,86].

The lipid–protein complex produced from damaged skin that is responsible for the severe immunosuppression linked to significant cutaneous burns is believed to be bound and denatured by cerium. Additionally, cerium nitrate hardens burn eschar, which is supposed to inhibit bacterial entry and maintain a moist wound. A broad-spectrum antibacterial agent, silver is efficient against yeast, bacteria, fungus, and viruses. When taken at the right concentration, it has also been demonstrated to be effective against vancomycin-resistant enterococci and Methicillin-resistant *Staphylococcus aureus* (MRSA) [81,82,83,84,85,86]. Additionally, silver is believed to lessen wound irritation and speed up recovery. The local wound environment affects the amount of silver required to have a bacteriostatic or bacteriocidal impact. It has shown to be effective against pathogens on the surface, but it might not have an impact on bacteria that have travelled a long way into the wound bed. So, when colonisation or critical colonisation is detected, silver may be utilised to assist in lowering the bacterial count in mild wound infections.

### 3.6. Stem Cell-Based Therapy

A promising new method in the area of regenerative medicine is stem cell-based therapy. The ability of stem cells to self-renew and specialise into distinct cell types is essential for physiologic tissue renewal and regeneration after injury, and is of great interest to biologists. Adult mesenchymal stem cells, embryonic stem cells, and, the more recently studied, induced pluripotent stem cells are the main sources of stem cells that are used for skin regeneration and wound healing among the several types of stem cells [57]. In essence, keratinocytes are produced from stem cells found in the skin’s basal layer, which differentiate for three to six weeks before becoming corneocytes, which in turn create the stratum corneum layer. Keratin, a protein that plays a significant structural role in the stratum corneum, is one of the many proteins that keratinocytes generate. Along with proteins, the stratum corneum’s complex lipid and cell membranes act as a significant barrier against bacteria and dehydration. The migration of fully developed cuboidal basal keratinocytes with large nuclei, phospholipid membranes, and organelles starts from the basal layer after roughly every 28 days [80,81,82,83,84,85,86,87].

A greater build-up of keratin and lipids develops throughout this turnover process and proceeds through terminal differentiation to form a stratum corneum. The dermo-epidermal junction, a basement membrane that separates the epidermal and dermal layers, is where the hemidesmosomes, which serve as cell adhesion molecules, are attached. Keratinocytes from the basal layer, ECM components, basal lamina, filaments, and anchoring fibrils make up the intricate dermo-epidermal junction structure. In order to reduce the possibility of the epidermis separating from the dermis layer, the dermo-epidermal junction must be restored during the wound healing process. The stem cell-based therapy for chronic wound healing makes use of a variety of processes, including growth factor interactions and activities, inflammation control, and immune process stimulation, for speeding vascularisation and re-epithelialisation. The ability of stem cell-based wound therapy to produce pro-regenerative cytokines and growth factors to encourage skin regeneration during the treatment of chronic wounds is largely responsible for its therapeutic potential [88,89].

### 3.7. Herbal Alternatives Acting as Activators for Wound Healing Factors

Wound healing may benefit greatly from a variety of plants or chemicals derived from plants that contain high concentrations of antioxidants and have anti-inflammatory, immunomodulatory, and antibacterial characteristics. Antioxidants help tissues recover from injury and can speed up wound healing. The antioxidant activity of flavonoids, anthraquinones, and naphthoquinones is strong. Shikonin, alkanin, lawsone, emodin, epigallocatechin-3-gallate, ellagic acid, and a few herbal extracts have strong antioxidant effects by scavenging ROS, preventing lipid peroxidation, and boosting intracellular antioxidant enzyme activities such as superoxide dismutase, catalase, and glutathione peroxidase [3,8]. Additionally, herbal medication encourages angiogenesis, fibroblast cell growth, and the production of temporary ECM.

Herbal extracts and other natural products’ immunomodulatory and anti-inflammatory properties hasten the healing of wounds. It is true that plants, and the chemical substances derived from them, aid in healing and treatment. Some herbal remedies stimulate the expression of VEGF and TGF-β, both of which are crucial in stimulating angiogenesis, granulation tissue development, and the deposition of collagen fibres. Other herbal medicines used in wound dressings act as inhibitors of the expression of the proteins TNF-α, IL-1, and inducible nitric oxide synthase, resulting in the induction of antioxidant and anti-inflammatory properties during different stages of the wound healing process. In the healing of cutaneous wounds, curcumin promotes the proliferation of fibroblasts, the development of granulation tissue, and the deposition of collagen [14,15]. Cinnamon bark has some therapeutic characteristics, including anti-inflammatory, anti-diabetes, anti-ulcer, anti-microbial, and hypoglycaemic effects. It can also help with diabetic and infected wounds. In addition to the above-mentioned qualities, cinnamon is known to contain considerable amounts of polyphenols, which may improve an animal’s ability to absorb glucose. Cinnamaldehyde, 2-hydroxycinnamaldehyde, and quercetin are anti-inflammatory properties of cinnamon components that can speed up wound healing. *Aloe vera* gel’s antioxidant qualities, which are attributable to certain components including indoles and alkaloids, have positive benefits on the healing of wounds [90]. Burns, ulcers, and surgical wounds remain as first-line conditions for *Aloe vera* treatment. Numerous organic bioactive substances, such as pyrocatechol, saponins, acemannan, anthraquinones, glycosides, oleic acid, phytol, and simple and complex water-soluble polysaccharides, are found in *Aloe vera*. *Aloe vera* has a variety of wound-healing mechanisms, most of which are attributable to raising the level of lysyl oxidase and the turnover rate of the collagen in the tissue. IL-1, IL-1, IL-6, TNF-α, PGE2, and nitrous oxide are only a few of the proinflammatory mRNAs that are transcribed when acemannan, a primary mucopolysaccharide from *Aloe vera*, is consumed. Reactive oxygen species and endogenous mitogen inhibitors are bound and captured by mesoglycan molecules, which facilitates phagocytosis.

Glycans prolong the activity of released cytokines, growth factors, and other bioactives. Through the action of cyclin D1 and AKT/mTOR signal pathways, topically administered acemannan has been shown to drastically shorten the time to wound closure in a rat wound healing model. It is well known that *Anethum graveolens* has antibacterial, anti-diabetic, and anti-inflammatory qualities that can speed up wound healing. Major constituents of dill essential oil include cis-carvone, limonene, phellandrene, and anethofuran. Burns, blisters, herpes, cuts, wounds, skin infections, and insect bites are just a few of the skin conditions that eucalyptus oil is traditionally used to treat [91,92,93]. *Securigerasecuridaca* is well known for its antibacterial properties, and helps infected wounds heal faster [94]. Antioxidant, anti-inflammatory, antidiabetic, cancer-preventive, antibacterial, antiviral, antimalarial, hypotensive, immunostimulatory, and hepatoprotective properties are all present in *Andrographis paniculata* extracts. In one study, it was found that treatment with a 10% aqueous leaf extract of *Andrographis paniculate* considerably improved the rate of wound closure in rats [95]. The polysaccharides diosgenin, yamogenin, gitogenin, tigogenin, and neotigogens are found in the seeds of *Trigonella foenum-graecum*. Saponins have steroidal actions that can reduce bodily inflammation. Fenugreek also contains mucilage, volatile oils, flavonoids, and amino acid alkaloids, which are bioactive components. 4-Hydroxyisoleucine is the other substance in fenugreek that is active. Fenugreek is said to release an anti-inflammatory chemical into the area of a cut, which reduces inflammation. Fenugreek’s antibacterial qualities may also enhance its anti-inflammatory effects. According to a study, the antibacterial characteristics of flavonoids and triterpenoids may help the wound healing process. Antioxidant properties of fenugreek are thought to hasten wound healing. The topical use of the fenugreek seed significantly increased the kinetics of wound contraction and epithelialisation [96].

In vivo studies have demonstrated that *Arctium lappa* root extract dramatically improves dermal ECM metabolism, affects glycosaminoglycan turnover, and minimises the appearance of wrinkles in human skin. *Arctium lappa* is also known to influence the Wnt/-catenin signalling pathway, which is recognised to be a significant regulator of wound healing, by regulating cell adhesion and gene expression in canine dermal fibroblasts. In diabetic rats, *Astragalus propinquus* and *Rehmanniaglutinosa* improve angiogenesis and reduce tissue oxidative stress to promote diabetic wound healing and post-ischaemic neovascularisation. In human skin fibroblasts, *Astragalus propinquus* and *Rehmanniaglutinosa* stimulated enhanced ECM deposition by activating the TGF-β signalling pathway. TNF-α and TGF-β1 levels were seen to be higher two days after the damage and to decrease as the wound healed. IL-10, on the other hand, was shown to be raised after 14 days, concurrent with wound healing [97]. Topical therapy with ethanolic *Ampelopsis japonica* increased re-epithelisation, granulation tissue development, vascularisation, and collagen deposition when compared to wounds treated with Vaseline or silver sulfadiazine.

Pharmacological benefits of *Angelica sinensis* include immunological modulator, anti-inflammatory, and anticancer properties. In human skin fibroblasts, extracts from Angelica sinensis have been demonstrated to promote cell proliferation, collagen secretion, and cell mobility while also activating an antiapoptotic mechanism. Additionally, extracts have been demonstrated to promote calcium fluxes and glycolysis, enhancing cell viability during tissue healing. Numerous studies have found conflicting results on *Angelica sinensis’* impact on the development of new blood vessels, raising questions about the plant’s role in angiogenesis [98]. By changing the expression of connective tissue growth factor (CTGF) and smooth muscle actin in vivo, extracts from the *Calendula officinalis* flower promote the formation of granulation tissue in excisional wounds of BALB/c mice. *Blumea balsamifera*, also known as kakoranda in Ayurveda, is used to cure rheumatism, coughs, fevers, and pains. Eczema, dermatitis, skin damage, bruising, beriberi, lumbago, menorrhagia, rheumatism, and skin damage are all treated using leaf extracts that are administered topically [99]. It has been discovered that homoisoflavonoids extracted from *Caesalpinia sappan* exhibit anti-inflammatory and antiallergic properties as well as the ability to block viral neuraminidase activity. The main catechin, (-)-epigallocatechin-3-gallate (EGCG), promotes keratinocyte growth and differentiation. By altering TGF-signalling, lowering MMP-1 and MMP-2 expression, and reducing the synthesis of type I collagen in human dermal fibroblasts, EGCG inhibits TGF- receptors. These characteristics suggested that EGCG may have anti-scarring capabilities [100].

*Entada phaseoloides* are high in saponins and tannins and are used topically to treat skin lesions as well as analgesic, bacteriocide, haemostatic, and anticancer agents. In diabetic cutaneous wounds, vitamin E controlled inflammation and oxidative stress. Additionally, vitamin E enhanced the anti-oxidative enzymes superoxide dismutase (SOD), glutathione peroxidase (GPX), and catalase (CAT) that are in charge of purging ROS and oxidised macromolecules from damaged tissues. By encouraging the growth of new blood vessels as well as re-epithelialisation, matrix deposition, and collagen synthesis, vitamin E had positive impacts on the wound healing process’ later stages (the proliferation and remodelling phases) [101]. Vasodilatation, blood lipid regulation, reduced inflammation; antioxidant, anti-cancer, anti-bacterial, anti-allergic, anti-ageing, and immunomodulatory potential are all benefits of *Panax ginseng* [102].

## 4. Localised Delivery Systems for Wound Healing

### 4.1. Microspheres/Microcarriers

A sort of injectable scaffold is a microsphere, often known as a microcarrier. Microspheres offer enough room for cell development and have good surface area-to-volume ratios. To further improve the transport of cells and bioactive compounds, microspheres with functional architectures (such as hollow or core-shell) can be easily customised and manufactured. As a result, functional microspheres have received a lot of attention recently as a new kind of injectable scaffold. Microspheres are typically referred to as spheres with a diameter between 1 and 1000 m. The diameter of most cells when they adhere and spread on a biomaterial is greater than 20 μm; hence, small-sized microspheres (<20 μm in diameter) are not suited as cellular carriers. Microspheres should therefore be between 20 and 200 μm in size when used as an injectable biomaterial for tissue engineering [103]. The development and uses of functional microspheres, including macroporous microspheres, nanofibrous microspheres, hollow microspheres, core–shell structured microspheres, and surface-modified functional microspheres, are covered after a brief introduction to the biomaterials and techniques for microsphere fabrication. Li and Wang explained the perspectives and directions for functional microspheres as injectable cell carriers, which are offered as a final step in the advancement of tissue regeneration [104].

Li et al. provide an explanation for the developments in functional microspheres, including the kinds of biomaterials used to make microspheres, the manufacturing processes for functional microspheres, and the uses of functional microspheres for the regeneration of bone, cartilage, the dentin–pulp complex, neural tissue, cardiac tissue, and skin. The use of microspheres has substantially increased as a result of the addition of additional structures and functions [105]. The core–shell configuration, for instance, can be easily used to combine cells in a microsphere with a regulated growth factor supply. Within a microsphere, there are numerous areas for cell migration and proliferation due to the hollow structure and macropores on the surface. Additionally, by incorporating nanofibrous architecture into microspheres, an ECM-like microenvironment is created that can direct tissue regeneration. This replicates the structure of natural ECM. Before using functional microspheres from a bench to a patient’s bedside, however, there is still considerable work to be undertaken.

The following description highlights some of the major obstacles to the creation of functioning microspheres [91,92,93,94,95,96,97]. In order to include biomimetic elements in functional microspheres, newer methods must first be developed. Phase separation is currently the only efficient way to make nanofibrous microspheres. While electrospinning and electrospray are used to create nanofibrous microparticles, their sizes and geometries are not well regulated. The traditional layer-by-layer self-assembly technique has trouble producing spheres smaller than a micrometre. Although one of the most efficient ways to create functional microspheres is using microfluidics, it is still difficult to create nanofibrous microspheres with this technology. More bio-inspired functional microspheres will be developed as a result of new fabrication methods that use modern microsphere fabrication techniques [103]. The exact control of bioactive chemical release from microspheres is also required. Currently, the majority of microsphere systems release bioactive molecules over a period of several days to a week, which is insufficient for the regeneration of many tissues. Additionally, there is a significant initial burst release from microspheres, and limiting the burst release is essential to enhancing therapeutic efficacy. The first burst release from functional microspheres is predicted to be tuned and reduced using methods and procedures including novel chemical and physical interaction factors in addition to traditional production parameters, such as polymer concentration and cross-linking time. The third one concerns the mechanical capabilities of microspheres [104].

The majority of microspheres are made from polymeric materials and typically have a porous structure; hence, their compressive moduli are quite low. Even though the mechanical strength is increased by adding inorganic elements such as hyaluronic acid, the microspheres still cannot be employed in load-bearing situations. In order to broaden the use of functional microspheres, research is being conducted to improve the mechanical properties of microspheres. Fourth, more evidence is required to support microspheres’ in vivo stability. To avoid diffusion of the microspheres to nearby faulty areas, microspheres are typically chemically cross-linked [105,106].

### 4.2. Inorganic Nanoparticles

Nanomaterials have received a lot of recent attention because of their improved efficacy and broad-spectrum antibacterial potential. Numerous metallic and metal oxide nanoparticles, such as Ag, Fe, Cu, and Au, as well as TiO_2_, ZnO, and Fe_3_O_4_, are being thoroughly researched for the treatment of infectious disorders. Considering that both of their counterparts are typical of low molecular weight, the two most popular systems of metal nanoparticles (NPs), gold (AuNPs) and silver (AgNPs) provide excellent methods for delivering pharmaceuticals. These nanoparticles have advantages over commonly used antibiotics because of their distinctive features, including size, shape, surface charge, dispersion, and chemical composition. Additionally, these synthetic antibiotics frequently only work against a certain type of bacterium or bacterial family, rendering them useless against a diverse range of bacterial species [107]. Currently, antibiotic efficiency has decreased mostly as a result of uncontrolled, excessive dosage, and prolonged use, which has favoured the establishment of multi-drug-resistant bacterial strains or “superbugs” such as MRSA-3. Nanomaterials have received a lot of recent attention because of their improved efficacy and broad-spectrum antibacterial potential.

Additional benefits of these materials include targeted drug delivery, solubility, improved cellular internalisation, tissue/cell selectivity, compatibility with tissue/tumour imaging, minimal adverse effects, etc. Due to their effectiveness in combating bacteria, silver nanoparticles have received the most attention among all those studied. However, little is known about the methods by which these nanoparticles destroy pathogens. It has been suggested that Ag^+^ ion release occurs from nanoparticles. However, the low stability and extreme cytotoxicity of the majority of silver nanoparticle (AgNP) forms limit their applicability in mammalian cells.

AgNPs must be engineered with various compositions to improve their properties and make them suitable for therapeutic application in order to overcome their current constraints. If these nanoparticles pass the required tests by regulatory agencies for better antibacterial efficacy and minimum cytotoxicity for patients, they may prove to be effective antibiotic alternatives or may be used in conjunction with antibiotics [106,107,108]. The antibacterial activity of metallic nanoparticles has been increased by a number of methods, including encasing silver nanoparticles in micelles, covering silver nanoparticles in gold, and capping gold nanoparticles with 5-amindole or sodium borohydride N-heterocyclic molecules.

A bimetallic nanoparticle with silver and gold was recently developed. Complex carbohydrates on the caps of these bimetallic nanoparticles improve their stability and other characteristics. These bimetallic NPs can be used for in vivo antimicrobial activities since they have considerably better antibacterial activities and do not harm mammalian cells. The use of silver to improve wound healing dates back to ancient times when it was utilised as an ion. Unlike silver ions (Ag^+^) or other forms of silver, which are used as nanoparticles, silver metal (Ag) has no known medical applications. They can impede the healing process since many bacteria, viruses, fungi, and even yeast is cytotoxic to them. This ion was used as part of a wound dressing that contained silver ions specifically to treat severe wounds [109]. In general, silver has numerous beneficial qualities, including broad-spectrum antibacterial activities. Silver-based lotions and other ointments are also available, and silver nanoparticle products can be used in a variety of therapeutic ways to help accelerate wound regeneration. The use of silver as a common bandage has been linked to decreased inflammation foci and scarring, possibly preventing bacterial development, and boosting the healing process, and possibly enhancing remodelling in the wound area, according to multivariate retrospective analyses. Therefore, using nanocomposites that are submerged in silver molecules improves the healing process by directly expressing collagen and certain growth factors that result in re-epithelialisation, neovascularisation, and the deposition of collagen fibres. Additionally, silver nanoparticles can cause the fibroblast to differentiate into myofibroblast, which is in charge of contracting the wound and quickening the healing process, and in a similar way, they can cause keratinocytes to be stimulated, proliferate, and move to the necessary area. For better wound healing, silver nanoparticles encourage keratinocyte migration from the edge into the core of the lesion. According to certain researchers, antimicrobial peptide–AgNP composite has been examined for its ability to speed up the remodelling process without having any negative effects on the dermal tissues. Resonance scattering dark-field microscopy makes use of gold nanoparticles to detect microbial cells and their byproducts, bio-image tumour cells, identify receptors on their surface, and analyse endocytosis [110].

Due to their chemical characteristics, optical stability, and simplicity of surface modification, gold nanoparticles (AuNPs) have been researched for medicinal applications such as wound healing. Before using gold nanoparticles for wound healing, they must fuse or have their surfaces modified with other biomolecules. For instance, the effectiveness of AuNPs to promote healing is increased by the addition of polysaccharide peptides. The application of gold nanoparticles to skin wounds boosted angiopoietin, VEGF, and collagen expression while decreasing mitomycin P and TGF-β1 levels [111]. It demonstrated decreased bacterial load and aided in recovery. Due to reduced blood circulation, systemically administered antibiotics may have trouble reaching injured skin tissue, rendering them ineffective for lowering bacterial numbers in granulation wounds. Recently, there has been a lot of fascinating study on the antibacterial properties of AuNPs, which makes them appropriate for possible co-use with antibiotics. Particularly in the 18 wt% composite group, the substance accelerated wound closure by increasing angiogenesis and fibroblast proliferation without inducing cell damage. According to a recent ex vivo permeation study, AuNPs can be effective in the treatment of burns as well since they can speed up the healing process and prevent microbial colonisation while being transdermally active [112].

### 4.3. Hydrogel

Insoluble hydrophilic materials known as hydrogels are created using synthetic polymers such as poly (methacrylates) and polyvinyl pyrrolidone. Complex hydrophilic organic cross-linked polymers called hydrogels have a base that is 80–90% water. These hydrogels can provide water to the wound site and aid in keeping it moist, which promotes quicker wound healing. These are created into contact lenses, drug delivery systems, wound dressings, electrodes, and sensors. These gels can be found as fixed flexible sheets or free-flowing amorphous gels. They have a limited capacity for fluid absorption through swelling, but they can also contribute moisture to a dry wound, aiding in autolytic debridement and maintaining a moist, thermally insulated wound environment [113].

Additionally, they have been demonstrated to increase granulation and epithelialisation, lower wound bed temperature, and have a relaxing and cooling effect. They have been shown to be a less efficient bacterial barrier than occlusive dressings and are permeable to gas and water. These dressings are primarily used to moisten dry wound beds and to soften and remove slough and necrotic wound debris. Due to their high-water concentration, they are unable to absorb substantial drainage; they absorb very slowly and are consequently useless on bleeding wounds; and they typically require a secondary dressing. They can be applied to a range of wounds, including vascular ulcers, pressure ulcers, and partial and full-thickness wounds [114]. Maceration is a potential problem since the skin around open wounds must be shielded from excessive moisture.

Hydrogels can be used in conjunction with topical drugs or antibacterial agents, which is one of their advantages. Infected wounds should not be treated with hydrogels in their fixed state. Hydrogels must be coated with additional dressings and left on for up to three days. They transfer oxygen and moisture vapour, but the type of secondary dressing employed affects how permeable they are to bacteria and fluids. Until an equilibrium condition is attained, these systems may swell in water and keep their original shape. The process of hydration, which is related to the presence of chemical groups such as -OH, -COOH, -CONH_2_, and -CONH-, as well as the existence of capillary regions and variations in osmotic pressure, is one of the interactions that contribute to the water sorption by hydrogels [115]. These are hydrophilic polymer networks that can absorb 10–20% of their dry weight in water as well as thousands of times that amount. These can dissolve and deteriorate or they can be chemically durable. When the polymer networks are bound together by molecular entanglements and/or secondary forces such as ionic and H-bonding, they are referred to as “reversible” or “physical” gels. When hydrogels have covalently bonded networks, they are referred to as “permanent” or “chemical” gels [116].

High-intensity radiation, freeze–thaw, or chemical processes can all be used to create hydrogels. Radiation, such as gamma rays, electron beams, X-rays, or ultraviolet light, is thought to be the most suited way for the creation of hydrogels since it allows for simple processing control and eliminates the need for potentially dangerous initiators or cross-linkers. Additionally, sterilisation and possible formation are both possible with irradiation. However, the mechanical strength of the hydrogels produced using this approach is subpar. Today, hydrogels are made using a freeze–thaw process to give them good strength and stability without the need for additional cross-linkers and initiators. The hydrogels’ limited swelling and thermal stability, as well as their opaque appearance, are the principal drawbacks of freeze–thawing. The application of hydrogel appears to considerably stimulate wound healing as compared to the standard gauze therapy. In order to create hydrogel wound dressings, a variety of natural and synthetic polymers with good biocompatibility are used [117].

There are several different types of hydrogels, including interpenetrating polymeric hydrogels, copolymer hydrogels, multipolymer hydrogels, and homopolymer hydrogels. In contrast to copolymer hydrogels, which are created by the cross-linking of two co-monomer units, one of which must be hydrophilic, homopolymer hydrogels are cross-linked networks of a single type of hydrophilic monomer unit. The cross-linking of more than three monomers results in the formation of multipolymer hydrogels. Finally, the swelling of a first network in a monomer and the reaction of the latter to generate a second intermeshing network structure results in interpenetrating polymeric hydrogels. It has been established that combining a natural polymer with a synthetic polymer appears to be a successful way to create materials with the necessary mechanical and thermal properties. It is also a quick process for producing the right forms, such as films, sponges, and hydrogels, to make a variety of biomedical devices.

For instance, chronic non-healing wounds are known to have an environment that is highly alkaline, whereas the healing process is more effective in an acidic environment. Therefore, manufactured dermal patches that can measure the pH of the wound continuously are essential for guiding point-of-care treatments and monitoring the healing process of chronic wounds. Since they are porous and permeable to oxygen and gas, hydrogels offer a lot of promise for use in biomedicine. They may be suggested as materials for both burn skin treatment and wound dressings. The hydrogel membrane was created based on a polyvinyl alcohol hydrogel, which may absorb wound exudates and release water, medications, or biomolecules (such as growth factors or antibiotics), creating the ideal environment for the healing of wounds. The epidermal sensor can also measure the ambient temperature, which allows it to deliver useful biological data regarding the state of the wound [118,119,120].

### 4.4. Vesicles Delivery System

Vesicular systems, which can be further divided into liposomes, ultra-deformable liposomes, and ethosomes, are composed of amphiphilic molecules because they have polar or hydrophilic regions and non-polar or lipophilic regions. It has been demonstrated that vesicular systems, including liposomes, niosomes, transferosomes, penetration enhancer-containing vesicles, and ethosomes, can improve the therapeutic activity of medications used to treat wounds. They can lengthen the shelf life of hydrophilic and hydrophobic medications and lessen major side effects including skin irritation, and act as a depot for controlled drug release. They can also improve the penetration of such medications into the skin. The two types of vesicular systems are hard vesicles, such as liposomes and niosomes, and flexible or ultra-flexible vesicles, such as transferosomes.

According to reports, rigid vesicles are ineffective for transdermal drug delivery because they stay on the stratum corneum’s outer layer and do not thoroughly penetrate the skin. After topical administration, liposomes can cause a variety of reactions. The majority of efforts have been concentrated on the topical treatments’ antibacterial action, which has fallen short due to the rising rate of antibiotic resistance. They can inhibit systemic absorption, maximise side effects, and provide a localising impact as well as tailored distribution to skin appendages. They can also improve drug deposition within the skin at the site of action. Additionally, these vesicles were crucial in the healing of wounds [121]. Antibiotics entrapped in liposomes exhibit reduced toxicity and more target specificity along with increased efficacy in treatment of bacterial infections and thus improve its pharmacokinetics and pharmacodynamics. Increased action against external pathogens that are resistant, as well as increased activity against intracellular pathogens, is also an attractive feature. Due to their occlusive action on the stratum corneum, lipid nanoparticles may be more appropriate in burn wounds and chronic wounds since they can prevent transepidermal water loss and maintain the lesion moisture. Additionally, compared to vesicular systems, nanostructured lipid carriers are offered as superior nano-delivery methods. They have great stability, low toxicity, high drug-loading capacity, and sustained drug release, which helps speed up wound healing and cuts down the number of drugs administered [122].

#### 4.4.1. Conventional Liposomes in Wound Healing

Because each pathophysiology differs, distinct skin wounds may require different treatments. According to the extent of the burn, acute burn wounds not only cause harm to the skin’s structures but also to all of the body’s systems because of the leakage of plasma into interstitial spaces. Additionally, the compromised skin barrier makes people more vulnerable to bacterial infections. In order to maintain skin functionality, it is crucial to prevent infections and promote re-epithelialisation while treating burn wounds. The majority of efforts have been concentrated on the topical treatments’ antibacterial action, which has fallen short due to the rising rate of antibiotic resistance. In order to combat infections and promote skin regeneration in burn wounds, a variety of nanosized lipid-based delivery systems, including liposomes, transferosomes, ethosomes, and lipid nanoparticles, have been investigated. The results are encouraging [122,123].

New treatments for chronic wounds have been made possible by advancements in vesicular drug delivery systems, which have decreased the cost, toxicity, and number of applications while enhancing the half-life and bioavailability of the medications. The lowered membrane permeability of madecassoside, a highly powerful substance used to heal wounds, was likewise outperformed by liposome encapsulation. A formulation with a high entrapment efficiency, excellent long-term stability, and small particle size was discovered. Furthermore, double-emulsion liposomes enhance transdermal penetration and wound healing, despite the fact that liposomes are non-toxic, biodegradable, and skin-compatible. Curcumin and quercetin have also been included in the liposomes. Polyphenols quercetin and curcumin have antioxidant and anti-inflammatory properties that are helpful for wound healing [124].

#### 4.4.2. Ultra-Deformable Liposomes or Transferosomes in Wound Healing

As a result of the creation of new vesicular systems called ultra-deformable liposomes, elastic vesicles, or transferosomes, conventional liposomes are currently used less frequently as transdermal delivery systems. Vesicles’ suppleness allows them to deform and enter skin pores that are much smaller than their diameters as intact vesicles. Ultra-deformable liposomes enter undamaged skin and penetrate deeply, allowing the systemic circulation to absorb them. Here, a transdermal hydration gradient allows the ultra-deformable liposomes to pass through the intact stratum corneum and enter the epidermis. This is a result of the vesicles’ high degree of deformability, which is brought on by the presence of surfactants, also referred to as “edge activator” molecules. With the ability to solubilise and fluidise epidermal lipids, edge activators have a significant impact on the exceptional deformability of transferosomes and increase their permeability capacity. Recently, consideration has been given for their capacity to traverse the stratum corneum among vesicular transferosomes. Transferosomes can transmit low bioavailability medications via the skin, according to numerous studies. Due to their deformability and ability to resist dry environments, transferosomes can pass to deeper skin layers undamaged. Transferosomes are therefore typically used for transdermal medication delivery in addition to their potential for topical distribution due to their capacity to penetrate deeply through epidermal layers and reach systemic circulation without the risk of vesicle rupture [121,123].

#### 4.4.3. Ethosomes and Phytosomes in Wound Healing

Drugs that are both extremely hydrophobic and highly hydrophilic now have easier access to the deep skin layers thanks to ethosomes. Depending on how much ethanol is present, the ethosomes can range in size from 103 to 200 nm. The second generation of liposomes can be introduced as ethosomes. As a result, the vesicular systems’ storage stability is a major challenge. However, ethosomes have more stability than regular liposomes. Several studies have looked into the utility of ethosomes in the treatment of wounds and have found that ethosomes transfer active ingredients to the skin more effectively than liposomes or conventional formulations. Recent research has shown that natural ingredients and antibacterial agents are more effective when they are encapsulated while treating burn wounds. In a different study, the ability of ethosomes loaded with silver sulfadiazine, a topical antibiotic regarded as the gold standard in burn wounds, to speed up the healing process and decrease bacterial infections in second-degree burns, was examined in vivo and in vitro. High entrapment efficiency is offered by phytosomes, which can be employed to distribute phytoconstituents topically for wound healing. The preparation was discovered to be risk-free and to have considerable antioxidant and wound-healing properties [123,124,125].

### 4.5. Emulsifying Drug Delivery System

The oral distribution of such medications is frequently linked to inadequate bioavailability, considerable intra- and inter-subject variability, and a lack of dose proportionality. Approximately 40% of novel drug candidates have poor water solubility. The use of surfactants, lipids, permeation enhancers, micronisation, salt formation, cyclodextrins, nanoparticles, and solid dispersions are just a few of the formulation strategies that have been used to tackle these issues. Recently, lipid-based formulations have received a lot of attention, with a focus on self-emulsifying drug delivery systems to increase the oral bioavailability of lipophilic medications. The fact that self-emulsifying drug delivery systems offer a significant interfacial area for the partitioning of the medication between oil and water is another benefit they have over straightforward oily solutions. Therefore, these systems may provide an improvement in the pace and amount of absorption as well as more consistent plasma concentration profiles for lipophilic medicines with dissolution-limited oral absorption [126].

By combining water and the non-ionic surfactant Tween 20 (Polysorbate 20), Ghosh et al. created a cinnamon oil microemulsion. Oil and surfactant were consumed in a 1:4 ratio. The microemulsion was discovered to be kinetically stable and generated with droplets that were around 5.79 nm in diameter. Ponto et al. studied that a microemulsion with antibacterial properties promotes wound healing in Wistar rats. In order to increase the solubility and bioavailability of hydrophobic/lipophilic medicines such as curcumin, self-emulsifying drug delivery systems are crucial substitute vehicles. Self-emulsifying drug delivery systems are physically stable isotropic mixes of oil/lipids, surfactants, and co-surfactants/co-solvents that have a great deal of potential as therapeutic drug delivery systems. To achieve the required therapeutic objectives, the self-emulsifying drug delivery systems formulations represent the hydrophobic drug in a solubilised state (nanoglobules) in the target location [127]. Through a number of methods, including drug solubilisation, droplet size reduction, improved membrane permeability, and protection of pharmaceuticals from chemical and enzymatic degradation, the self-emulsifying drug delivery systems formulations can increase drug bioavailability.

Different self-emulsifying drug delivery systems formulations were created and tested with an eye toward skin applications, including self-emulsifying drug delivery systems for the topical delivery of mangosteen peel and self-emulsifying drug delivery systems for the transdermal drug delivery of curcumin, *Opuntia ficusindica* fixed oil, *Piper cubeba* essential oil, and *Opuntia ficusindica* fixed oil (*Garcinia Mangostana* L.). The absorption of self-emulsifying drug delivery systems by the fibroblasts was found to be linearly dependent and dose-dependent. When curcumin is synthesised into nanocarriers, which have the least amount of cytotoxicity when compared to free drug solution, the cellular uptake of curcumin is facilitated [125,126,127,128].

### 4.6. Nanofiber/Film/Membrane

One of the most significant areas of science and technology in the twenty-first century that can be likened to the industrial revolution is nanotechnology. Soon, nanotechnology will have a substantial impact on the global economy and industry, as well on as the technology used by humans and their way of life. In the past, different materials such as animal fat, plant fibres, honey, etc., have been used to cover wounds (Table 2). Polymers have been employed in several research studies to make films for use as wound dressings. With two external dimensions that are identical in size at the nanoscale (about 100 nm) and a third dimension that is noticeably larger, nanofibres are one of the most fascinating classes of nanomaterials. Depending on the medication’s solubility in the polymer solution, a polymer solution (polymer + particular solvent) is first created, after which a predetermined proportion of the drug is added, resulting in either a homogenous solution or a suspension [126,127,128,129,130,131]. This combination is electrospun to create nanofibres that contain a solid polymer–drug complex.

A wide range of polymers can be used to create nanofibres. For dressing nanofibres, there are only three types of polymers now available: natural polymers, synthetic polymers, and mixed polymers. Natural polymers are appropriate for use in biomedical applications due to their wide range of benefits, including biocompatibility, non-toxicity, biodegradability, antibacterial properties, and desirable mechanical structure. In the procedure, the solvent evaporates. On the other hand, nanofibres are made of a polymeric base, which makes up most of the fibre’s composition, and a bioactive molecule (such as a protein, hormone, or medication), or another type of polymer, but in a lesser amount than the base polymer. Currently, the three primary techniques for producing nanofibres are electrospinning, the phase-separation method, and the self-assembly method. The method that produces nanofibres most frequently is electrospinning. Depending on the electrospinning technique employed, various types of nanofibres can be produced. Today, the commercialisation of electrospinning equipment is advancing quickly. The most popular electrospinning methods include bubble electrospinning, melt electrospinning, coaxial electrospinning, self-bundling electrospinning, and nano-spider electrospinning [132,133,134,135,136,137,138,139,140,141,142,143,144]. Fibre electrospinning processes can be used to create polymer nanofibres (50–1000 nm) (wet or hot melt electro-spinning). Numerous excellent characteristics of nanofibres include their substantial surface area, the potential for surface functionalisation, tunable porosity, a broad range of material options, and outstanding mechanical performance. The advantageous characteristics of electrospun nanofibres, such as mechanical stability, high porosity, high surface area to volume ratio, and ability to exchange water, oxygen, and nutrients, encourage good cell attachment, differentiation, and proliferation. They enable the monitoring of infection and healing markers due to their capacity to mimic the structure and operation of ECM [145]. The nanofibres are excellent candidates for a variety of biomedical applications, such as tissue-engineered scaffolds (such as skin, cartilage, bone, and blood vessels), dressings for wound healing, biomedical devices, biosensors, and drug delivery systems, because of their exceptional capabilities. Electrospun nanofibre mats offer a native extracellular matrix-like structure with high interconnected porosity (60–90%), great absorbencies, and a water absorbance capacity of 18–21% more than films made of the same polymers [146]. These characteristics, along with balanced moisture and gas permeability, create an environment that is suitable for preventing exogenous infection, cell migration and proliferation, haemostasis, exudate absorption, and cell respiration in wounds. Electrospun nanofibres can control the proliferation, migration, differentiation, and synthesis of native extracellular matrix in skin cells.

Tissue engineering and wound healing are two of the most important and intriguing biomedical uses of nanofibres. Nanofibres have been utilised in the treatment of diabetic ulcers and wounds to aid in wound healing, haemostasis, skin regeneration, and wound dressing. Nanofibre keeps the wound surface moist while healing because it can hold more moisture in its structure. As a result, the nanofibres cannot adhere to the surface of the wound. Additionally, the porous nanofibre network makes it simpler for oxygen to diffuse into the wound area. To remove toxins from the blood of individuals with kidney failure, wearable blood purification systems may integrate the nanofibre membrane. Nanofibre scaffolds hold great promise for wound healing. These scaffolds are used in the treatment of diabetic ulcers, skin rejuvenation, wound dressings to encourage healing, and haemostasis [147,148]. Nanofibres keep the wound area moist while it heals because of their capacity to hold more moisture within their structures. As a result, the scaffold cannot adhere to the damaged surface. Furthermore, oxygen may easily diffuse to the location of the wound thanks to the porous networks. Nanofibres have the power to regulate a range of skin cell responses, including proliferative, migratory, differentiating, and extracellular matrix accumulation. Their microstructure ideally complements the ECM structure, which encourages cellular proliferation, adhesion, and growth. While creating a larger surface area than bulk materials, nanofibre arrangements have increased porosity, which is advantageous for cell activities.

The polymers used to create scaffolds for wound dressing include collagen, poly-vinyl pyrrolidone, polyacrylic acid, polyvinyl alcohol, gelatin, chitosan, silk fibroin, polyesters, and poly-urethane [149]. The goal of a wound dressing is to quickly achieve haemostasis, and it should also have strong antibacterial properties to guard against bacterial infections from the environment. The ability of electrospinning to construct nanofibrous membranes for wound dressings that can provide a moist environment surrounding the wound region to facilitate healing has drawn a great deal of interest. In order to create composite nanofibre membranes that carry a reservoir of biogenic AgNPs for use as a wound dressing, Bardania et al. used *T. polium* extract as a reducing agent. This method of “green synthesis”, which does not use external stabilisers or reducing agents, produced AgNPs quickly, cheaply, and effectively [76,82,109].

### 4.7. Foam Dressings

Foam dressings are permeable to both gases and water vapour and have a polyurethane foundation. The outer layer’s hydrophobic qualities shield it from liquids while allowing gaseous exchange and the passage of water vapour. Silicone-based rubber foam, or silicone, conforms to the shape of wounds. Depending on the thickness of the wound, foam can absorb varied amounts of wound drainage. There are foam dressings that are both adhesive and non-adhesive. Lower leg ulcers, mild to heavily exuding wounds, and granulating wounds can all benefit from foam dressings. They offer both thermal insulation and excellent absorption thanks to their hydrophilic characteristics [153]. These incredibly adaptable dressings should be used on moderate-to-heavy exudative wounds, partial- and full-thickness wounds that are granulating, or slough-coated donor sites, ostomy sites, mild burns, and diabetic ulcers. Due to their capacity to cause wounds to become even drier, they are not advised in dry or eschar-covered wounds and vascular ulcers. Due to their high absorbency and moisture vapour permeability, they are typically utilised as main dressings for absorption, and supplementary dressings are not necessary. The disadvantage of foam dressings is that they need to be changed frequently and are not appropriate for low exudating wounds, dry wounds, or dry scars since they need exudates to heal. They can stay in place for up to 4 to 7 days, but once they become saturated with exudates, they need to be changed. When removed, they are non-traumatic due to their makeup. They can also be applied to infected wounds if they are changed daily [39]. Numerous papers have examined various foam dressing forms, mostly because of their propensity for liquid and/or permeability to moisture vapour. These characteristics may make some foam more suitable for treating weakly exudative wounds as opposed to lesions that drain excessively [154]. The primary limitation of this kind of dressing is that it requires a second dressing, such as an elastic bandage or a film, to adhere to the wound. Foam dressings are used for deep wounds that have produced a lot of exudates as well as for long-term wounds such as venous ulcers. They may also be applied under compression bandages [149,153].

### 4.8. Biological Dressings

In order to restore the wound healing process and incorporate active biological agents to assist the wound healing process, advanced biological therapies are currently emerging in ischaemic wound therapies. The use of active biological agents, such as plant-derived active biomolecules with antioxidant, antibacterial, or anti-inflammatory properties, may be used in biological wound-healing therapies, which aim to aid the restoration of the body’s natural repair mechanisms. Biological dressings stop contamination, heat loss, protein and electrolyte loss, and evaporative water loss [15,22]. Bioactive wound dressings can be created using naturally occurring biomaterials with endogenous activity or materials that release bioactive chemicals. Chitin, chitosan, hydrocolloids, alginate, and derivatives of organic biopolymers are a few examples of these biomaterials. Biological dressings made from animal collagen are known as collagen dressings. The fibroblast-produced protein collagen plays a part in each step of wound healing. The extracellular matrix is primarily made up of collagen, which gives it strength [29]. This category includes dressings made from collagen sourced from bovine, porcine, or avian sources. These products are all intended to hasten the healing process. Protease activity is decreased by both collagen and oxidised regenerated cellulose (ORC), which is usually coupled with collagen. Proteases have a variety of roles in the healing of wounds, albeit their levels typically decline over time. There is some proof that collagen dressings for venous leg ulcers are at the very least comparable to other modern wound dressings [36].

A glycoaminoglycan component of the ECM, hyaluronic acid has distinct biological and physical properties. Like collagen, hyaluronic acid is biocompatible, biodegradable, and naturally immune-suppressive. During the proliferative stage of wound healing, chitosan encourages the development of granulation tissue. Biological dressings are said to be more effective than other forms of dressings when compared to other dressings [37,38]. Alginates and chitosan have also been used successfully in a haemostatic dressing.

In a rat full-thickness wound model, it was demonstrated that sponges based on carboxymethyl chitosan, alginates, and a Chinese medication could quickly induce haemostasis and sustain wound closure. Lyophilisation and spray-drying procedures were used to create multi-resorbable haemostatic dressings made of chitosan, sodium/calcium alginate, and/or carboxymethyl cellulose. This kind of treatment was initially applied as a biological dressing to cover extensive burn wounds. Additionally, it has been used to heal chronic wounds, such as venous stasis ulcers [39]. Allografts from cadavers serve as a substrate for the formation of granulation tissue. The cadaveric skin is attached, an antibiotic/antimicrobial dressing is placed over the graft, and a compression dressing is used to lessen swelling once the wound bed has been prepped as previously mentioned. To enhance the physical and biological properties of tissue engineering products, such as wound dressings, chemical cross-linking is frequently utilised in the manufacturing process. Sodium trimetaphosphate, a secure and non-toxic cross-linking agent, can be used to improve the functional qualities of various polysaccharides, including starch, cellulose, and xanthan [48]. In patients with an ischaemic wound, biological wound dressing or topical agent therapy may hasten wound healing, increase limb salvage, be reasonably priced, and offer potential safety with nontoxic low-risk therapy. Therefore, patients with ischaemic wounds should also receive local wound care using biological dressings as an adjuvant therapy. To support the effectiveness and long-term results of these biological dressings in patients with ischaemic wounds, additional randomised studies are required [49].

### 4.9. Charcoal Dressings

By absorbing gases produced by bacteria, activated charcoal dressings serve the primary purpose of reducing wound odour. They can absorb odour molecules due to their huge surface area and function as a deodorising agent. Wound odour is very subjective in nature since it is challenging to define and quantify. Leg ulcerations and diverse fungating lesions are the wounds most frequently linked to odour generation. Numerous aerobic bacteria as well as anaerobes including *Bacteroides* and *Clostridium* species are among the organisms usually linked to malodorous wounds [51,52,53]. Eliminating the problematic organism is the best strategy for treating wound smells. Antibiotics taken systemically may be successful, but it may be challenging to obtain an adequate concentration of the drug at the infection site. In numerous studies to date, topical treatments such as metronidazole, clindamycin, honey, and sugar have demonstrated promise in this area. Activated charcoal dressings are frequently used in malodorous wounds; however, odour control has not received as much attention in the literature as wound healing has. According to current clinical experiences, it is evident that these dressings can reduce wound odour, but there are not any hard facts on just charcoal ingredients [56,57,58].

### 4.10. Three-Dimensional Skin Substitutes

Recently, several tissue engineering technologies have become available; these technologies take a fundamentally unique and new approach. Among these, three-dimensional free-form fabrication—often referred to as three-dimensional bioprinting—offers several benefits over traditional skin tissue engineering. An innovative method for designing and engineering human organs and tissues is three-dimensional bioprinting, a flexible automated on-demand platform for the free-form production of complex living constructions. Here, we use human skin as a representative example to show the potential of three-dimensional bioprinting for tissue engineering. In order to simulate the epidermis, dermis, and dermal matrix of the skin, keratinocytes, fibroblasts, and collagen were employed as constituent cells [154]. This method has enormous potential for the creation of three-dimensional skin tissue since it can dispense living cells, soluble components, and phase-changing hydrogels in a desired pattern while preserving very high cell viability.

The biomimetic mechanical cues that support vascularisation, alignment of fibrous proteins in the ECM, integration of dermal and epidermal components, and adhesion between these layers are absent from the existing skin grafts and their production techniques. When the biomechanics of the repaired tissue at the wound site are compared to those of the healthy tissue nearby wounds, this problem may become more difficult. Therefore, to prevent the separation of the layers during application, a regenerative skin scaffold should take biomimetic mechanical cues into account. By adjusting the physicomechanical characteristics of each layer, the scaffold can offer tailored microenvironments for various cell types [155]. The use of autologous epidermal sheets as a kind of skin replacement has progressed into the use of more sophisticated bilayered cutaneous tissue-designed skin substitutes. However, their regular use for restoring normal skin anatomy is constrained by insufficient vascularisation, rigid drug/growth factor loading, and the inability to regenerate skin appendages such as hair follicles [156].

Recent developments in cutting-edge science from stem cell biology, nanotechnology, and other vascularisation techniques have given researchers a huge head start in creating and modifying tissue-engineered skin substitutes for better skin regeneration and wound healing. The creation of scaffolds for tissue engineering, films and membranes for wound healing, artificial tissues, and even artificial organs, all include the use of three-dimensional printing. Superior flexibility, regulated porosity, and reproducibility are the key benefits of the scaffolds made using this method. One of the current synthetic alternatives imitates the layer of skin made up of keratinocytes and fibroblasts on the collagen matrix. Only the dermal components with fibroblasts on the collagen matrix are present in the cellular matrix. Focused application, which stimulates wound healing and directs the healing of severe burns without scars, yielded encouraging results.

Bioengineered organisms can adjust to their surroundings and release the growth factors and cytokines used in dressings. Both venous leg ulcers and diabetic foot ulcers can be treated using bioengineered dressings. The additive manufacturing method of three-dimensional bioprinting offers a viable method for creating biocompatible artificial skins by carefully layering growth factors, biomaterials, and living cells on top of one another. This automated technology is a versatile tool that, in terms of accuracy and usefulness, is ideal for therapeutic usage. Through the exact deposition of many cells and biomaterials, the bioprinted skin analogues closely replicate the native skin’s architecture and heterogenicity [157]. The bioprinted skin constructions need to meet several requirements in terms of their compositional and functional qualities. The ability to transmit nutrients and wound exudates should be the first feature of bioprinted skin substitutes. The ability to precisely deposit various skin cells, such as keratinocytes, fibroblasts, adipocytes, melanocytes, Langerhans cells, etc., at certain layers and places is another requirement for bioprinted analogues. Finally, the bioprinted structure needs to be strong, biocompatible, biodegradable, and able to withstand the external forces and pressures that are present under in vivo circumstances. Despite revolutionising wound treatment, skin replacements have several drawbacks. They necessitate lengthy cell culture processes that extend production times. Most of the vascularisation is inadequate. Scarring occurs at the graft margins, which exacerbates existing functional and aesthetic issues. Infection is always a possibility. Since they only have fibroblasts and keratinocytes, they are unable to develop differentiated structures including sweat and sebum glands, hair follicles that cycle, pigmentation, sensory innervation, and motor innervation, as well as the epidermal barrier and dermal–epidermal junction [39,55,56,57,58,59,60,61,62,63,64,65,66,67,68,69,70,71,72,73,74,75,76,77,78,79,80,81,82,83,84,85,86,87,88,89,90,91,92,93,94,95,96,97,98,99,100,101,102,103,104,105,106,107,108,109,110,111,112,113,114,115,116,117,118,119,120,121,122,123,124,125,126,127,128,129,130,131,132,133,134,135,136,137,138,139,140,141,142,143,144,145,146,147,148,149,150,151,152,153,154,155].

Porosity, degradation, and mechanical properties should closely resemble the native skin structure. Onto a scaffold made of hyaluronic acid in three dimensions, autologous cultured fibroblasts are sown. Apligraf, a skin substitute for venous ulcers made from keratinocytes and fibroblast-seeded collagen, has received FDA approval [156,157]. Commercially available skin substitutes include IntegraTM artificial skin, which is made of a collagen/chondroitin-6-sulphate matrix coated with a thin silicone sheet, and AllodermTM, which is made of normal human fibroblasts with all cellular components removed. LaserskinTM, BiobraneTM, BioseedTM, and Hyalograft3-DTM are a few further alternatives.

### 4.11. Dendrimers

Dendrimers are a class of nanoscale (1–100 nm) three-dimensional globular macromolecules with numerous arms branching out from a central core. They have unique structural characteristics, including high levels of branching, multivalency, globular architecture, and well-defined molecular weight, making them promising drug delivery scaffolds. The distinctive structure of dendrimers makes them an ideal nanomaterial for the administration of medications to target certain tissues or molecules with solubility challenges. This is performed by trapping the drugs inside of their void spaces, branches, or outside functional groups. An expanding field of research involves cross-linking collagen with functionalised nanoparticles to produce scaffolds for use in wound healing. Due to their spherical structure, dendrimers can engage via hydrogen bonding, lipophilicity, and charge interactions with tiny medicines, metals, or imaging moieties that can fit within their branches. In addition to their structure, dendrimers may be the best drug delivery vehicles for many therapeutic treatments because of their size and lipophilicity, which allow them to easily permeate cell membranes. These distinguishing qualities have increased interest in using dendrimers nanoparticles for wound healing in research [158].

In order to build an efficient nanoparticle-mediated scaffold for tissue engineering and wound healing applications, Vedhanayagam et al. showed that nanoparticle form is a critical component that needs to be investigated. The re-epithelialisation and collagen deposition in damaged tissue are accelerated more quickly by the spherical shape of zinc oxide triethoxysilane poly(amidoamine) dendrimer generation 1 nanoparticles than by other shapes. The highly branched 3D structures known as antimicrobial peptide dendrimers (AMPDs) have a central core and a high density of flexible surface groups for possible molecule attachment. Their capacity to penetrate the cell membrane and the presence of functional groups of amino acid residues are what cause them to have bactericidal effects [159].

Mannose-decorated globular lysine dendrimers have the ability to reduce inflammation by targeting and reprogramming macrophages to the M2 phenotype, which is characterised by significant mannose receptor clustering on the cell surface and the elongated shape; increased production of TGF-1, IL-4, and IL-10; decreased secretion of IL-1, IL-6, and tumour necrosis factor (TNF); and increased ability to induce fibroblast proliferation. These results show that M2 macrophage polarisation can be directed by mannose-decorated globular lysine dendrimers, which may be helpful in the treatment of injuries and inflammation [160].

The development of an antisense delivery method based on dendrimers has assisted in the development of an antisense therapy strategy to treat bacterial infections. Dendrimers themselves might function as powerful antibacterial substances. In contrast, those with metal cores can produce active antimicrobial agents such as metal ions and ROS, which can kill bacteria. For example, those with positively charged surfaces typically have significant interactions with negatively charged bacterial cell membranes. AgNPs may benefit from the alternate template that dendrimers can offer. Dendrimers have a stronger antibacterial action when combined with silver than silver has by itself. In computerised tomography or immune-sensor coatings, poly-(amido-amine) dendrimers have been utilised as contrast agents. These highly branched dendritic molecules have a restricted size range, a well-defined globular structure, and a relatively large molecular mass [161].

Among the family of monodisperse, highly branched units with a clearly defined structure are dendrimers such as poly(amidoamine). Their surface contains a cationic primary amine group, which enables them to participate in chemical bonding. For the treatment of wounds, poly-(amido-amine) dendrimers are primarily used as a vector for the transport of various hydrophilic or hydrophobic medicines and genes. The hesperidin-loaded dendrimer was found to be biocompatible and suitable for use in wound healing after a haemolysis investigation [162]. Tumour suppression applications have demonstrated the anti-angiogenic properties of polycationic dendrimers. Angiogenesis and neovascularisation, however, are important components for skin regeneration following serious burn wounds because newly created blood vessels aid in the healing process by improving nutrition and oxygen supply to regenerating tissues. Therefore, using polycationic antimicrobial peptide dendrimers could prevent angiogenesis and endanger the process of skin regeneration [163].

Dendrimers are incorporated into gelatin nanofibres through covalent conjugation, which not only increases the capacity of nanofibre construction for drug loading but also offers a great deal of versatility for creating multipurpose electrospun dressing materials. Since dendrimer–gelatin nanofibre constructs are designed to treat a variety of wounds, including chronic wounds, burns, and skin malignancies, they can be customised to offer cutting-edge therapies [164,165]. Dendrimers have a variety of uses in biomedicine, but their toxicity has also been described in order to analyse the restrictions on their use. The structure of a chemical determines its toxicity as well as all of its other characteristics. A dendrimer’s toxicity can either be increased or decreased depending on specific components (core, branch, and surface groups) [158,161].

### 4.12. Carbon Nanotubes

The carbon nanotubes are a class of stiff, stable, hollow nanomaterials with a variety of special physical, chemical, and mechanical properties that have been widely used as catalyst supports, nanowires, electronic components, and more recently in the fields of biomedical engineering and medical chemistry [166]. A needle-like structure with a significant surface area, carbon nanotubes are an allotropic form of carbon with nanoscale dimensions and µm lengths. The carbon atoms join to form sheets of graphite, which is made up of six-membered carbon atom rings. Graphite then spirals into tubes. Carbon nanotubes can be divided into single-wall and multiwall varieties based on the number of graphite layers present. CNTs have tensile strengths up to 63 gigapascals, which is around 50 times stronger than steel, and elastic moduli between 1.0 and 1.8 terapascals. With the goal of boosting drug delivery, regulating drug release, and improving therapeutic activity, carbon nanotubes are now being functionalised by various pharmacologically active compounds, with some degree of success [167]. The degradation products of carbon nanotubes can be eliminated by the functional tissues of the human body, making them non-toxic and safe for consumption [168]. The fibroblasts, which are crucial to the cell renewal system and the healing process of open wounds, may come into contact with the carbon nanotubes employed in the biomedical field and found in the environment as they pass through the skin or open wounds [166]. Proteins and receptors found in cell membranes can cling tightly to carbon nanotubes. Carbon nanotubes and cells must adhere to one another [166].

The ability of organic compounds with a carbon component to improve the antibacterial activity of polymers is well known. Because they can function as a conductive bridge over the insulating bilayer, carbon nanotubes’ antimicrobial activity can be increased following functionalisation with -OH and -COOH functional groups or hybridisation with metallic compounds. After being incorporated into chitosan biopolymers, multiple-walled carbon nanotubes boost cell survival and proliferation with few negative side effects. Using the solution casting technique, Liu et al. created a bio-nanocomposite film-based wound healing dressing. The film’s ability to absorb water is crucial to preventing tissue dehydration, limiting the growth of microorganisms, and safeguarding wound maceration. It is anticipated that the multiple-walled carbon nanotubes’ hydrophilic nature and water-holding capacity will increase the bio-nanocomposite film’s ability to absorb water. Through chemical interactions, the presence of multiple-walled carbon nanotubes increases ROS formation [169]. The physical properties of fibrous proteins found in the ECM, such as collagen and elastin, are approximated by the greater diameter of multiple-walled carbon nanotubes compared to single-walled carbon nanotubes.

According to molecular research conducted by Khalid et al., bacterial cellulose functionalised with multiwalled carbon nanotubes displayed a lower than control level of pro-inflammatory cytokines IL-1 and TNF- and a higher level of VEGF expression, which may have favoured a quicker healing process [170]. Carbon nanotubes may pass through a variety of physiological barriers and have substantial tissue penetration. Isoniazid/chitosan/carbon nanotube nanoparticles were created by Chen et al. and have been shown to dramatically speed up the healing of tuberculosis ulcers [171]. Experimental tests demonstrated by Zhao et al. revealed that the antibacterial gel made of nano-silver and multiple empty carbon nanotubes has a superior anti-infective impact on burn wounds and can significantly lessen the frequency of dressing changes [172]. By affecting cell spreading, adhesion, migration, and survival, exposure to carbon nanotubes causes inflammation, genotoxicity, and inhibits dermal fibroblasts’ capacity to heal wounds. While concurrently harming the cytoskeleton and upsetting actin stress fibres in NIH 3T3 murine fibroblasts and human dermal fibroblasts, multiwalled carbon nanotubes reduced DNA synthesis and the levels of adhesion-related genes. Skin allergies are made worse, and keratinocyte cytotoxicity is also produced, by low doses of topical multiwalled carbon nanotubes [166,167,168,169].

### 4.13. Microneedle Drug Delivery Systems

The efficacy of traditional single-drug therapies is subpar, and penetration depth limits the effectiveness of drug delivery [173]. In the realm of wound healing, microneedle dressings with transdermal drug delivery capabilities have been crucial [174]. Additionally, the microstructure of microneedles allows for efficient medication administration to the target location while preventing overly strong skin and patch adherence. Additionally, temperature-sensitive hydrogel has been used to encapsulate vascular endothelial growth factor (VEGF) in the chitosan microneedle array micropores. As a result, the temperature increase brought on by the inflammatory response at the site of wounds can be used to controllably achieve the smart release of the medications [175]. The adaptable approach of the microneedle patch has been presented and has achieved several outstanding successes in the fields of disease therapy, biosensing, skin vaccination, and wound healing. Microneedles can efficiently deliver the desired active pharmaceuticals due to their better loading capacity and well-designed microstructures when compared to those used in conventional drug delivery systems. However, the microneedle that is so frequently used today is typically made from synthetic polymer materials that were created by difficult chemical synthesis using harsh experimental processes and environmentally hazardous organic reagents. This raises the danger of side effects. Additionally, the development of microneedle-based iatrotechnics is constrained by the acquisition of loaded active pharmaceuticals typically through a period of brutal elimination and extremely stringent clinical studies [176]. Wang et al. created a three-dimensional origami microneedle patch with extremely tiny needle structures, microfluidic channels, and numerous functionalities that was said to be able to detect biomarkers, distribute medications in a controlled manner, and monitor motions to speed up wound healing [177]. Exosomes can only partially reach the injury site through passive diffusion, according to Liu et al.’s explanation of the potential of microneedle delivery of exosomes for transdermal application [178]. As a result, the therapeutic effects and clinical applications are significantly diminished. An antibacterial and angiogenesis-promoting double-layer microneedle patch was reported by Gao et al. for the treatment of diabetic wounds. Tetracycline hydrochloride, an antibacterial medication filled with hyaluronic acid, serves as the tip of the double-layer microneedle, while deferoxamine, an angiogenic medication, serves as the substrate [179]. The transdermal route has been used to administer bioactives using microneedles. The bioactives can pass through the epidermis with the assistance of these microneedle devices. Microneedles are created via in situ polymerisation utilizing a mould-based approach using biomaterials such as chitosan, hyaluronic acid, and maltose [180]. In the realm of wound management, new individualised and programmable microneedle wound dressings are extremely valuable. With its straightforward, efficient, and safe qualities, the microneedle-mediated drug delivery system can also offer a novel method for the treatment of diabetic wounds. It has a wide range of applications in relevant biomedical sectors. The low stiffness of the microneedles for insertion into human skin poses a challenge for the translation of this method. The constraints and high cost of microfabrication technology have restricted the development of microneedle-based systems utilising standard subtractive techniques [181,182].

## 5. Future Prospects

The alignment of fibrous proteins, cell type, ECM composition, water content, and mechanical qualities vary between skin layers. However, the skin’s natural ability to regenerate is constrained to treating relatively small wounds, necessitating the use of topical medications in some or most cases. Therefore, because the healing process entails complicated events to re-establish functional tissues following injury, big skin wounds present a problem. Due to the existence of several cells and chemicals orchestrating a process, wound healing has always been the most difficult problem. Any problem may hinder the healing process and cause an acute wound to become chronic. The difficulty in keeping immune cells in their naive immune state is currently a major barrier to their incorporation in the skin, and it is probably due to their ineffective incorporation within the skin strata as well as the absence of other functionally significant cells. Adult stem cells have emerged as a promising therapy for promoting scarless wound healing among the numerous techniques that have so far been used in the treatment of skin ulcers. Mesenchymal stem cells have drawn more attention than other adult stem cells due to their capacity for immunomodulation and tissue regeneration. Intense research is still being conducted on the creation of novel and efficient therapies for wound care. Future topical therapy for wound care can be evaluated using comparative effectiveness research as a technique. Over the past ten years, a number of novel techniques and items have been developed, helping to meet the ongoing demand for advancements in wound care. Numerous other techniques, including hyperbaric oxygen, growth hormones, biologic dressings, skin substitutes, and regenerative materials, have also demonstrated effectiveness in speeding up the healing of wounds.

## Figures and Tables

**Table 1:** Commonly used antimicrobial agents with target species.

Class	Name	Wound Dressing Material	Tested Strains	Administration	References
Macrolides	Clarithromycin Erythromycin	PVA hydrogels	*Pseudomonas aeruginosa* *Staphylococcus aureus*	Oral/Systemic/ Topical/ ophthalmic	[58]
Tetracycline	Tetracycline Chlortetracycline	Cotton fabric coated with chitosan-Poly (vinyl pyrrolidone)-PEG	*E. coli* *S. aureus*	Oral/Topical	[59]
		Scaffolds with Collagen Microsphere with gelatin	*E. coli* *S. aureus*	Oral/Systemic/ Topical	[61]
Aminoglycosides	Streptomycin Neomycin	Wafers and film based on polymer Polyox/carrageenan	*E. coli* *S. aureus* *P. aeruginosa*	Oral/Topical/Systemic oral/topical	[66] [67]
		poly(styrene sulfonic acid-co-maleic acid) (PSSA-MA)/polyvinyl alcohol (PVA) ion exchange nanofibres	*E. coli* *S. aureus*		
Fluoroquinolones	Norfloxacin Ciprofloxacin	Films and nanofibre mats of povidone Electrospun fibers based on thermoresponsive polymer poly(N-isopropylacrylamide), poly(L-lactic acid-co-ɛ-caprolactone) Hydrogels from 2-hydroxyethyl methacrylate/citraconic anhydride-modified collagen Films and nanofibre mats of povidine	*E. coli* *Bacillus subtilis* *E. coli* *S. aureus* *S. aureus* *E.coli* *Bcillus subtilis*	Topical Topical Topical	[69] [70] [71]

**Table 2:** Localised delivery systems of several drugs.

Formulation	Drug	Administration	Outcome	References
Nanofibre	Gentamicin sulphate (GS)	Topical application	It promotes cell adhesion and proliferation to scaffolds, and ultimately tissue regeneration and promotes healing process.	[129]
Nanofibre	Ferulic acid	Topically applied every day	Increased migration of cells to the wound site to fill the gap and increased proliferation causing rapid wound healing.	[130]
Nanofibre	Berberine	Topical treatment	Exhibited antibacterial activity against Gram-positive and Gram-negative bacterium. Animal studies on the STZ-induced diabetic rats demonstrated that the CA/Gel/Beri dressing enhanced the wound healing process.	[131]
Nanofibre	Peppermint	Topical dressing	Accelerated response and less inflammation and nanofibres showed potent wound healing activity for diabetic ulcers.	[132]
Nanofibre	Beta-glucan (βG)	Topically applies once a day	Enhanced maturation of granulation tissue and better healing process.	[133]
Nanofibre	Huangbai liniment (compound phellodendron liquid, CPL)	Topical treatment	It was also found that composite nanofibre membrane could reduce wound inflammation, down-regulate the expression of IL-1β and TNF-α inflammatory genes, and facilitate wound healing.	[134]
Nanofibre	Gentamicin salt (GEN)	Topical dressing	It has excellent antibacterial properties against Gram-negative E. coli, which is due to the unique properties of silver nanoparticles for antibacterial activity, and this composite has a good release profile for wound healing.	[135]
Nanofibre	Poly (caprolactone) (PCL)	Topical dressing once every day	It significantly promoted adhesion, proliferation and induced angiogenesis, collagen deposition, and re-epithelialisation in the wound sites of diabetic mice model, as well as inhibited inflammation reaction.	[136]
Hydrogel	Curcumin	Daily topical treatment	Shorten inflammatory process, prevents infection and re-epithelisation and promotes wound closer.	[137]
Liposome	Citicoline/chitosan	Topical treatment	Chitosan-coated liposomes containing citicoline have emerged as a potential approach for promoting the healing process in diabetic rats. However, the therapeutic effectiveness of the suggested approach in diabetic patients needs to be investigated.	[138]
Liposome	Curcumin	Topically applied once a day for 18 days	Curcumin-loaded liposomes in lysine–collagen hydrogel was found to be the most effective of the three formulations in promoting wound healing. Hence, this formulation can serve as a prototype for further development and has great potential as a smart wound dressing for the treatment of surgical wounds.	[139]
Liposome	DangguiBuxue	Topically applied	Remarkably accelerates wound closure, enhances hydroxyproline content in wound granulation tissue, promotes cutaneous wound healing by reducing the inflammatory response and improving fresh granulation tissue formation, and significantly increases the density of blood vessels and cell proliferation.	[140]
Nanoparticle	Silver	Topical treatment	Rapid healing and improved cosmetic appearance via reduction in wound inflammation and modulation of fibrogenic.	[141]
Nanoparticle	Zinc oxide (ZnO_2_)	Topical dressing	Had excellent anti-bacterial activity and rapid wound healing.	[142]
Nanomembrane	Triphala		Triphala PCL shows good broad spectrum of antimicrobial activity and biocompatibility and helps control wound infection and enhanced healing due to antioxidants of Triphala.	[143]
Nanomembrane	Chitosan	Topical treatment	This nanomembrane serves as an excellent microenvironment for cell adhesion, migration, proliferation, and differentiation. An in vivo experiment with this nanomembrane was also conducted, showing that it has a great capability for stem cell delivery for skin tissue reconstruction.	[144]
Hydrogel	Glycosaminoglycan	Topical application	Promotion of tissue proliferation and regeneration of vascular vessels.	[138]
Deformable liposome	Curcumin	Daily topical treatment	CDLs in hydrogel preserved hydrogel’s bioadhesiveness to a higher degree than both NDLs and ADLs. In addition, CDLs-in-hydrogel enabled the most sustained skin penetration of curcumin and hence facilitates wound healing.	[145]
Liposomal ointment	Retinoic acid and growth factors	Topical application	Liposomal ointment on deep partial-thickness burn model stimulated wound closure (*p* < 0.001), promoted skin appendage formation and increased collagen production, thus improving healing quality.	[146]
Hydrogel nanoparticle	Copper (Cu)	Topical treatment	CuNP-comprised hydrogels exhibited a significant decrease in bacterial activity and promoted effective wound closure with negligible toxicity in our histological evolution.	[147]
Nanogel	Cerium oxide	Topical application	Showed significant antibacterial properties even at low absorptions and is effective at damage and scar production.	[148]
Nanoparticle	Thrombin	Topical treatment	The proportionate improvement in skin tensile strength after treatment with bound thrombin suggests that the novel thrombin conjugates may lessen surgical difficulties.	[149]
Microneedle	Trichostatin A, histone deacetylase 4	Topical	The microneedle-mediated Trichostatin A patch has been shown to improve the healing of diabetic wounds by reducing inflammation, promoting tissue regeneration, and inhibiting histone deacetylase 4.	[150]
Metal–organic framework microneedle patch	Nitric oxide	Topical application	Delivering nitric oxide molecules more precisely and deeply into the wound site may be made possible by the integrated microneedle’s porous shape, increased specific surface area, and enough mechanical strength.	[151]
Microneedle	Curcumin nanodrugs/new Indocyanine Green/hyaluronic acid	Topical treatment	The two-layered microneedles platform has the potential to be used as a competitive technique for the treatment of melanoma since it can simultaneously remove the tumour and speed up wound healing.	[152]

## Data Availability

Not applicable.

## Abbreviations

AgNPs: Silver nanoparticles; AMPDs: Antimicrobial peptide dendrimers; AuNPs: Gold nanoparticles; CAT: Catalase; CNTs: Carbon nanotubes; CTGF: Connective tissue growth factor; ECM: Extracellular matrix; EGCG: Epigallocatechin-3-gallate; EGF: Epidermal growth factor; EGR1: Early-growth response 1; ELAM-1:Endothelial leukocyte adhesion molecule 1; FGF-2: Fibroblast growth factor -2; GPX: Glutathione peroxidase; HIF-1: Hypoxia-Inducible Factor; ICAM-1: Intercellular adhesion molecule 1; IGF-1: Insulin-like Growth Factor-1; IL-1: Interleukin-1; IRF5: Interferon regulatory factor 5; LOX-1: Lectin-like oxLDL (oxidised low-density lipoprotein); MAPK: Mitogen-activated protein kinase; MCP-1: Monocyte Chemoattractant Protein-1; MMP-1: Matrix metalloproteinase-1; MRSA: Methicillin-resistant *Staphylococcus aureus*; mTOR: mammalian target of rapamycin; ORC: Oxidised regenerated cellulose; PDGF: Platelet-derived growth factor; pNFκB: Phospho-Nuclear Factor Kappa B; PPAP: Polyprenylated acylphloroglucinol; pSTAT1:Phosphosignal transducer and activator of transcription 1; ROS: Reactive oxygen species; SMAD: Suppressor of Mothers against Decapentaplegic; SOD: Superoxide dismutase; TGF- β: Transforming Growth Factor-beta; TLR: Toll-like receptors; TNF-α: Tumour Necrosis Factor Alpha; VCAM-1:Vascular cell adhesion molecule 1; VEGF: Vascular endothelial growth factor; VPF: Vascular Permeability Factor.

## References

[1] Hassanshahi A., Hassanshahi M., Khabbazi S., Hosseini-Khah Z., Peymanfar Y., Ghalamkari S., Su Y.W., Xian C.J. (2019). Adipose-derived stem cells for wound healing. J. Cell. Physiol..

[2] Kim H.S., Sun X., Lee J.H., Kim H.W., Fu X., Leong K.W. (2019). Advanced drug delivery systems and artificial skin grafts for skin wound healing. Adv. Drug Deliv. Rev..

[3] Ryall C., Duarah S., Chen S., Yu H., Wen J. (2022). Advancements in Skin Delivery of Natural Bioactive Products for Wound Management: A Brief Review of Two Decades. Pharmaceutics.

[4] Martin P., Nunan R. (2015). Cellular and molecular mechanisms of repair in acute and chronic wound healing. Br. J. Dermatol..

[5] Han G., Ceilley R. (2017). Chronic Wound Healing: A Review of Current Management and Treatments. Adv. Ther..

[6] Yuan Z., Zhang K., Jiao X., Cheng Y., Zhang Y., Zhang P., Zhang X., Wen Y. (2019). A controllable local drug delivery system based on porous fibers for synergistic treatment of melanoma and promoting wound healing. Biomater. Sci..

[7] Elviri L., Bianchera A., Bergonzi C., Bettini R. (2017). Controlled local drug delivery strategies from chitosan hydrogels for wound healing. Expert Opin. Drug Deliv..

[8] Gorain B., Pandey M., Leng N.H., Yan C.W., Nie K.W., Kaur S.J., Marshall V., Sisinthy S.P., Panneerselvam J., Molugulu N. (2022). Advanced drug delivery systems containing herbal components for wound healing. Int. J. Pharm..

[9] Veith A.P., Henderson K., Spencer A., Sligar A.D., Baker A.B. (2019). Therapeutic strategies for enhancing angiogenesis in wound healing. Adv. Drug Deliv. Rev..

[10] Guo S., Dipietro L.A. (2010). Factors affecting wound healing. J. Dent. Res..

[11] Rubalskii E., Ruemke S., Salmoukas C., Aleshkin A., Bochkareva S., Modin E., Mashaqi B., Boyle E.C., Boethig D., Rubalsky M. (2019). Fibrin glue as a local drug-delivery system for bacteriophage PA5. Sci. Rep..

[12] Jang M.J., Bae S.K., Jung Y.S., Kim J.C., Kim J.S., Park S.K., Suh J.S., Yi S.J., Ahn S.H., Lim J.O. (2021). Enhanced wound healing using a 3D printed VEGF-mimicking peptide incorporated hydrogel patch in a pig model. Biomed. Mater..

[13] Shedoeva A., Leavesley D., Upton Z., Fan C. (2019). Wound Healing and the Use of Medicinal Plants. Evid. Based Complement. Altern. Med..

[14] Ibrahim N., Wong S.K., Mohamed I.N., Mohamed N., Chin K.Y., Ima-Nirwana S., Shuid A.N. (2018). Wound Healing Properties of Selected Natural Products. Int. J. Environ. Res. Public Health.

[15] Kumari A., Raina N., Wahi A., Goh K.W., Sharma P., Nagpal R., Jain A., Ming L.C., Gupta M. (2022). Wound-Healing Effects of Curcumin and Its Nanoformulations: A Comprehensive Review. Pharmaceutics.

[16] Fatehi P., Abbasi M. (2020). Medicinal plants used in wound dressings made of electrospun nanofibers. J. Tissue Eng. Regen. Med..

[17] Wang Y., Malcolm D.W., Benoit D.S.W. (2017). Controlled and sustained delivery of siRNA/NPs from hydrogels expedites bone fracture healing. Biomaterials.

[18] Barrientos S., Stojadinovic O., Golinko M.S., Brem H., Tomic-Canic M. (2008). Growth factors and cytokines in wound healing. Wound Repair Regen..

[19] Kolimi P., Narala S., Nyavanandi D., Youssef A.A.A., Dudhipala N. (2022). Innovative Treatment Strategies to Accelerate Wound Healing: Trajectory and Recent Advancements. Cells.

[20] Saghazadeh S., Rinoldi C., Schot M., Kashaf S.S., Sharifi F., Jalilian E., Nuutila K., Giatsidis G., Mostafalu P., Derakhshandeh H. (2018). Drug delivery systems and materials for wound healing applications. Adv. Drug Deliv. Rev..

[21] Wang W., Lu K.J., Yu C.H., Huang Q.L., Du Y.Z. (2019). Nano-drug delivery systems in wound treatment and skin regeneration. J. Nanobiotechnol..

[22] Farahani M., Shafiee A. (2021). Wound Healing: From Passive to Smart Dressings. Adv. Healthc. Mater..

[23] Kuffler D.P. (2016). Photobiomodulation in promoting wound healing: A review. Regen. Med..

[24] Chereddy K.K., Vandermeulen G., Préat V. (2016). PLGA based drug delivery systems: Promising carriers for wound healing activity. Wound Repair Regen..

[25] Sorg H., Tilkorn D.J., Hager S., Hauser J., Mirastschijski U. (2017). Skin Wound Healing: An Update on the Current Knowledge and Concepts. Eur. Surg. Res..

[26] Gantwerker E.A., Hom D.B. (2012). Skin: Histology and physiology of wound healing. Clin. Plast. Surg..

[27] Velnar T., Bailey T., Smrkolj V. (2009). The wound healing process: An overview of the cellular and molecular mechanisms. J. Int. Med. Res..

[28] Kaiser P., Wächter J., Windbergs M. (2021). Therapy of infected wounds: Overcoming clinical challenges by advanced drug delivery systems. Drug Deliv. Transl. Res..

[29] Boateng J.S., Matthews K.H., Stevens H.N., Eccleston G.M. (2008). Wound healing dressings and drug delivery systems: A review. J. Pharm. Sci..

[30] Broughton G., Janis J.E., Attinger C.E. (2006). Wound healing: An overview. PlastReconstr. Surg..

[31] Eming S.A., Martin P., Tomic-Canic M. (2014). Wound repair and regeneration: Mechanisms, signaling, and translation. Sci. Transl. Med..

[32] Ud-Din S., Bayat A. (2017). Non-animal models of wound healing in cutaneous repair: In silico, in vitro, ex vivo, and in vivo models of wounds and scars in human skin. Wound Repair Regen..

[33] Masoumpour M.B., Nowroozzadeh M.H., Razeghinejad M.R. (2016). Current and Future Techniques in Wound Healing Modulation after Glaucoma Filtering Surgeries. Open Ophthalmol. J..

[34] Cabourne E., Clarke J.C., Schlottmann P.G., Evans J.R. (2015). Mitomycin C versus 5-Fluorouracil for wound healing in glaucoma surgery. Cochrane Database Syst. Rev..

[35] Moghadam S.E., MoridiFarimani M., Soroury S., Ebrahimi S.N., Jabbarzadeh E. (2019). Hypermongone C Accelerates Wound Healing through the Modulation of Inflammatory Factors and Promotion of Fibroblast Migration. Molecules.

[36] Torregrossa M., Kakpenova A., Simon J.C., Franz S. (2021). Modulation of macrophage functions by ECM-inspired wound dressings—A promising therapeutic approach for chronic wounds. Biol. Chem..

[37] Chattopadhyay S., Raines R.T. (2014). Review collagen-based biomaterials for wound healing. Biopolymers.

[38] Vivcharenko V., Wojcik M., Palka K., Przekora A. (2021). Highly Porous and Superabsorbent Biomaterial Made of Marine-Derived Polysaccharides and Ascorbic Acid as an Optimal Dressing for Exuding Wound Management. Materials.

[39] Westby M.J., Dumville J.C., Soares M.O., Stubbs N., Norman G. (2017). Dressings and topical agents for treating pressure ulcers. Cochrane Database Syst. Rev..

[40] Berger M.M., Binz P.A., Roux C., Charrière M., Scaletta C., Raffoul W., Applegate L.A., Pantet O. (2022). Exudative glutamine losses contribute to high needs after burn injury. J. Parenter. Enter. Nutr..

[41] Guan Y., Niu H., Liu Z., Dang Y., Shen J., Zayed M., Ma L., Guan J. (2021). Sustained oxygenation accelerates diabetic wound healing by promoting epithelialization and angiogenesis and decreasing inflammation. Sci. Adv..

[42] Bossi F., Tripodo C., Rizzi L., Bulla R., Agostinis C., Guarnotta C., Munaut C., Baldassarre G., Papa G., Zorzet S. (2014). C1q as a unique player in angiogenesis with therapeutic implication in wound healing. Proc. Natl. Acad. Sci. USA.

[43] Jin L., Guo X., Gao D. (2021). R-responsive MXene nanobelts for wound healing. NPG Asia Mater..

[44] Vijayan V., Sreekumar S., Singh F., Govindarajan D., Lakra R., Korrapati P.-S., Kiran M.S. (2019). Praseodymium-Cobaltite-Reinforced Collagen as Biomimetic Scaffolds for Angiogenesis and Stem Cell Differentiation for Cutaneous Wound Healing. ACS Appl. Bio Mater..

[45] Ridiandries A., Tan J.T.M., Bursill C.A. (2018). The Role of Chemokines in Wound Healing. Int. J. Mol. Sci..

[46] Xiao T., Yan Z., Xiao S., Xia Y. (2020). Proinflammatory cytokines regulate epidermal stem cells in wound epithelialization. Stem Cell Res. Ther..

[47] Heinrich P.-C., Behrmann I., Haan S., Hermanns H.-M., Müller-Newen G., Schaper F. (2003). Principles of interleukin (IL)-6-type cytokine signalling and its regulation. Biochem. J..

[48] Mi F.L., Wu Y.B., Shyu S.S., Schoung J.Y., Huang Y.B., Tsai Y.H., Hao J.Y. (2002). Control of wound infections using a bilayer chitosan wound dressing with sustainable antibiotic delivery. J. Biomed. Mater. Res..

[49] Tamahkar E., Özkahraman B., Süloğlu A.K., İdil N., Perçin I. (2020). A novel multilayer hydrogel wound dressing for antibiotic release. J. Drug Deliv. Sci. Technol..

[50] Sabitha M., Rajiv S. (2015). Preparation and characterization of ampicillin-incorporated electrospun polyurethane scaffolds for wound healing and infection control. Polym. Eng. Sci..

[51] Ye S., Jiang L., Wu J., Su C., Huang C., Liu X., Shao W. (2018). Flexible amoxicillin-grafted bacterial cellulose sponges for wound dressing: In vitro and in vivo evaluation. ACS Appl. Mater. Interfaces.

[52] Basha M., AbouSamra M.M., Awad G.A., Mansy S.S. (2018). A potential antibacterial wound dressing of cefadroxil chitosan nanoparticles in situ gel: Fabrication, in vitro optimization and in vivo evaluation. Int. J. Pharm..

[53] Nikdel M., Rajabinejad H., Yaghoubi H., Mikaeiliagah E., Cella M.A., Sadeghianmaryan A., Ahmadi A. (2021). Fabrication of cellulosic nonwoven material coated with polyvinyl alcohol and zinc oxide/mesoporous silica nanoparticles for wound dressing purposes with cephalexin delivery. ECS J. Solid State Sci. Technol..

[54] Rădulescu M., Holban A.-M., Mogoantă L., Bălşeanu T.-A., Mogos-anu G.-D., Savu D., Popescu R.C., Fufă O., Grumezescu A.M., Bezirtzoglou E. (2016). Fabrication, Characterization, and Evaluation of Bionanocomposites Based on Natural Polymers and Antibiotics for Wound Healing Applications. Molecules.

[55] Bakadia B.M., Boni B.O.O., Ahmed A.A.Q., Zheng R., Shi Z., Ullah M.W., Lamboni L., Yang G. (2022). In Situ Synthesized Porous Bacterial Cellulose/Poly (vinyl alcohol)-Based Silk Sericin and Azithromycin Release System for Treating Chronic Wound Biofilm. Macromol. Biosci..

[56] Ciftci F., Ayan S., Duygulu N., Yilmazer Y., Karavelioglu Z., Vehapi M., ÇakırKoç R., Sengor M., Yılmazer H., Ozcimen D. (2022). Selenium and clarithromycin loaded PLA-GO composite wound dressings by electrospinning method. Int. J. Polym. Mater. Polym. Biomater..

[57] de Souza R.F.B., de Souza F.C.B., Moraes Â.M. (2016). Polysaccharide-based membranes loaded with erythromycin for application as wound dressings. Appl. Polym. Sci..

[58] Alavarse A.C., de Oliveira Silva F.W., Colque J.T., da Silva V.M., Prieto T., Venancio E.C., Bonvent J.J. (2017). Tetracycline hydrochloride-loaded electrospun nanofibers mats based on PVA and chitosan for wound dressing. Mater. Sci. Eng. C.

[59] Khampieng T., Wnek G.-E., Supaphol P. (2014). Electrospun DOXY-h loaded-poly(acrylic acid) nanofiber mats:In vitro drug release and antibacterial properties investigation. J. Biomater. Sci. Polym. Ed..

[60] Akota I., Alvsaker B., Bjørnland T. (1998). The effect of locally applied gauze drain impregnated with chlortetracycline ointment in mandibular third-molar surgery. Acta Odontol. Scand..

[61] Abbott P.V., Hume W.R., Pearman J.W. (1990). Antibiotics and endodontics. Aust. Dental. J..

[62] Michalska-Sionkowska M., Kaczmarek B., Walczak M., Sionkowska A. (2018). Antimicrobial activity of new materials based on the blends of collagen/chitosan/hyaluronic acid with gentamicin sulfate addition. Mater. Sci. Eng. C.

[63] Anjum A., Sim C.H., Ng S.F. (2018). Hydrogels containing antibiofilm and antimicrobial agents beneficial for biofilm-associated wound infection: Formulation characterizations and In vitro study. AAPS PharmSciTech.

[64] Ahire J.J., Robertson D.D., van Reenen A.J., Dicks L.M.T. (2017). Polyethylene oxide (PEO)-hyaluronic acid (HA) nanofibers with kanamycin inhibits the growth of Listeria monocytogenes. Biomed. Pharmacother..

[65] Nitanan T., Akkaramongkolporn P., Rojanarata T., Ngawhirunpat T., Opanasopit P. (2013). Neomycin-loaded poly (styrene sulfonic acid-co-maleic acid) (PSSA-MA)/polyvinyl alcohol (PVA) ion exchange nanofibers for wound dressing materials. Int. J. Pharm..

[66] Denkbaş E.U.R.B., Öztürk E., Özdem&unknownr N., Agalar C. (2004). Norfloxacin-loaded chitosan sponges as wound dressing material. J. Biomater. Appl..

[67] Contardi M., Heredia-Guerrero J.A., Perotto G., Valentini P., Pompa P.P., Spanò R., Goldonic L., Bertorelli R., Athanassiou A., Bayera I.S. (2017). Transparent ciprofloxacin-povidone antibiotic films and nanofiber mats as potential skin and wound care dressings. Eur. J. Pharm. Sci..

[68] Li H., Williams G.R., Wu J., Wang H., Sun X., Zhu L.M. (2017). Poly (N-isopropylacrylamide)/poly (l-lactic acid-co-ɛ-caprolactone) fibers loaded with ciprofloxacin as wound dressing materials. Mater. Sci. Eng. C.

[69] Pamfil D., Vasile C., Tarţău L., Vereştiuc L., Poiată A. (2017). pH-Responsive 2-hydroxyethyl methacrylate/citraconic anhydride–modified collagen hydrogels as ciprofloxacin carriers for wound dressings. J. Bioact. Compat. Polym..

[70] Pásztor N., Rédai E., Szabó Z.I., Sipos E. (2017). Preparation and Characterization of Levofloxacin-Loaded Nanofibers as Potential Wound Dressings. Acta Med. Marisiensis.

[71] Singh B., Dhiman A. (2015). Designing bio-mimetic moxifloxacin loaded hydrogel wound dressing to improve antioxidant and pharmacology properties. RSC Adv..

[72] Kurczewska J., Pecyna P., Ratajczak M., Gajęcka M., Schroeder G. (2017). Halloysite nanotubes as carriers of vancomycin in alginate-based wound dressing. Saudi Pharm. J..

[73] Amiri N., Ajami S., Shahroodi A., Jannatabadi N., Darban S.A., Bazzaz B.S.F., Pishavar E., Kalalinia F., Movaffagh J. (2020). Teicoplanin-loaded chitosan-PEO nanofibers for local antibiotic delivery and wound healing. Int. J. Biol. Macromol..

[74] Rolston K.V.I., Dholakia N., Ho D.H., LeBlanc B., Dvorak T., Streeter H. (1996). In-vitro activity of ramoplanin (a novel lipoglycopeptide), vancomycin, and teicoplanin against gram-positive clinical isolates from cancer patients. J. Antimicrob. Chemother..

[75] Habash M.B., Park A.J., Vis E.C., Harris R.J., Khursigara C.M. (2014). Synergy of silver nanoparticles and aztreonam against Pseudomonas aeruginosa PAO1 biofilms. Antimicrob. Agents Chemother..

[76] Jones R.N. (1989). Critical assessment of the newer non-quinolone oral antimicrobial agents. Antimicrob. Newsl..

[77] Bauernfeind A., Schweighart S., Chong Y. (1989). Extended broad spectrum β-lactamase in Klebsiella pneumoniae including resistance to cephamycins. Infection.

[78] Teaima M.H., Elasaly M.K., Omar S.A., El-Nabarawi M.A., Shoueir K.R. (2022). Wound healing activities of polyurethane modified chitosan nanofibers loaded with different concentrations of linezolid in an experimental model of diabetes. J. Drug Deliv. Sci. Technol..

[79] Mohammed A.A., Ali M.A., Ahmed O.S. (2018). To evaluate safety and efficacy of tedizolid phosphate in the management of several skin infections. Int. J. Res. Pharm..

[80] Dou J.L., Jiang Y.W., Xie J.Q., Zhang X.G. (2016). New is old, and old is new: Recent advances in antibiotic-based, antibiotic-free and ethnomedical treatments against methicillin-resistant Staphylococcus aureus wound infections. Int. J. Mol. Sci..

[81] Fajardo A.R., Lopes L.C., Caleare A.O., Britta E.A., Nakamura C.V., Rubira A.F., Muniz E.C. (2013). Silver sulfadiazine loaded chitosan/chondroitin sulfate films for a potential wound dressing application. Mater. Sci. Eng. C Mater. Biol. Appl..

[82] Hasselmann J., Kühme T., Acosta S. (2015). Antibiotic prophylaxis with trimethoprim/sulfamethoxazole instead of cloxacillin fails to improve inguinal surgical site infection rate after vascular surgery. Eur. J. Vasc. Endovasc. Surg..

[83] Gjorevski N., Nikolaev M., Brown T.E., Mitrofanova O., Brandenberg N., DelRio F.W., Yavitt F.M., Liberali P., Anseth K.S., Lutolf M.P. (2022). Tissue geometry drives deterministic organoid patterning. Science.

[84] ValadanTahbaz S., Azimi L., Asadian M., Lari A.R. (2019). Evaluation of synergistic effect of tazobactam with meropenem and ciprofloxacin against multi-drug resistant Acinetobacter baumannii isolated from burn patients in Tehran. GMS Hyg. Infect. Control.

[85] Yang M., Hu Z., Hu F. (2012). Nosocomial meningitis caused by Acinetobacter baumannii: Risk factors and their impact on patient outcomes and treatments. Future Microbiol..

[86] NourianDehkordi A., MirahmadiBabaheydari F., Chehelgerdi M., RaeisiDehkordi S. (2019). Skin tissue engineering: Wound healing based on stem-cell-based therapeutic strategies. Stem Cell Res. Ther..

[87] Gonzales K.A.U., Fuchs E. (2017). Skin and its regenerative powers: An alliance between stem cells and their niche. Dev. Cell.

[88] Azari Z., Nazarnezhad S., Webster T.J., Hoseini S.J., Brouki Milan P., Baino F., Kargozar S. (2022). Stem cell-mediated angiogenesis in skin tissue engineering and wound healing. Wound Repair Regen..

[89] Kamath J.V.C., Rana A.C., Chowdhury A.R. (2003). Pro-healing effect of Cinnamomum zeylanicum bark. Phytother. Res..

[90] Liang J., Cui L., Li J., Guan S., Zhang K., Li J. (2021). Aloe vera: A medicinal plant used in skin wound healing. Tissue Eng. Part B Rev..

[91] Teplicki E., Ma Q., Castillo D.E., Zarei M., Hustad A.P., Chen J., Li J. (2018). The Effects of Aloe vera on Wound Healing in Cell Proliferation, Migration, and Viability. Wounds.

[92] Hamman J.H. (2008). Composition and applications of Aloe vera leaf gel. Molecules.

[93] RaesiVanani A., Mahdavinia M., Kalantari H., Khoshnood S., Shirani M. (2019). Antifungal effect of the effect of *Securigera securidaca* L. vaginal gel on Candida species. Curr. Med. Mycol..

[94] Dai Y., Chen S.R., Chai L., Zhao J., Wang Y., Wang Y. (2019). Overview of pharmacological activities of Andrographis paniculata and its major compound andrographolide. Crit. Rev. Food Sci. Nutr..

[95] Nagulapalli Venkata K.C., Swaroop A., Bagchi D., Bishayee A. (2017). A small plant with big benefits: Fenugreek (*Trigonella foenum-graecum* Linn.) for disease prevention and health promotion. Mol. Nutr. Food Res..

[96] Chan Y.S., Cheng L.N., Wu J.H., Chan E., Kwan Y.W., Lee S.M., Leung G.P., Yu P.H., Chan S.W. (2011). A review of the pharmacological effects of *Arctium lappa* (burdock). Inflammopharmacology.

[97] Wei W.L., Zeng R., Gu C.M., Qu Y., Huang L.F. (2016). Angelica sinensis in China-A review of botanical profile, ethnopharmacology, phytochemistry and chemical analysis. J. Ethnopharmacol..

[98] Givol O., Kornhaber R., Visentin D., Cleary M., Haik J., Harats M. (2019). A systematic review of Calendula officinalis extract for wound healing. Wound Repair Regen..

[99] Li Y., Dong M., Wu Z., Huang Y., Qian H., Huang C. (2021). Activity Screening of the Herb Caesalpinia sappan and an Analysis of Its Antitumor Effects. Evid. Based Complement. Altern. Med..

[100] Sugimoto S., Matsunami K. (2018). Biological activity of Entada phaseoloides and Entada rheedei. J. Nat. Med..

[101] Kiefer D., Pantuso T. (2003). Panax ginseng. Am. Fam. Physician..

[102] Fang Q., Yao Z., Feng L., Liu T., Wei S., Xu P., Guo R., Cheng B., Wang X. (2020). Antibiotic-loaded chitosan-gelatin scaffolds for infected seawater immersion wound healing. Int. J. Biol. Macromol..

[103] Huang S., Lu G., Wu Y., Jirigala E., Xu Y., Ma K., Fu X. (2012). Mesenchymal stem cells delivered in a microsphere-based engineered skin contribute to cutaneous wound healing and sweat gland repair. J. Dermatol. Sci..

[104] Li H., Wang F. (2021). Core-shell chitosan microsphere with antimicrobial and vascularized functions for promoting skin wound healing. Mater. Des..

[105] Zhang D., Ouyang Q., Hu Z., Lu S., Quan W., Li P., Chen Y., Li S. (2021). Catechol functionalized chitosan/active peptide microsphere hydrogel for skin wound healing. Int. J. Biol. Macromol..

[106] Negut I., Grumezescu V., Grumezescu A.M. (2018). Treatment Strategies for Infected Wounds. Molecules.

[107] MofazzalJahromi M., SahandiZangabad P., MoosaviBasri S.M., SahandiZangabad K., Ghamarypour A., Aref A., Karimi M., Hamblin M.R. (2018). Nanomedicine and advanced technologies for burns: Preventing infection and facilitating wound healing. Adv. Drug Deliv. Rev..

[108] Likus W., Bajor G., Siemianowicz K. (2013). Nanosilver—Does it have only one face?. Acta Biochim. Pol..

[109] Chen C.Y., Yin H., Chen X., Chen T.H., Liu H.M., Rao S.S., Tan Y.J., Qian Y.X., Liu Y., Hu X.K. (2020). Ångstrom-scale silver particle-embedded carbomer gel promotes wound healing by inhibiting bacterial colonization and inflammation. Sci. Adv..

[110] Qiu L., Wang C., Lan M., Guo Q., Du X., Zhou S., Cui P., Hong T., Jiang P., Wang J. (2021). Antibacterial Photodynamic Gold Nanoparticles for Skin Infection. ACS Appl. Bio. Mater..

[111] García I., Henriksen-Lacey M., Calvo J., de Aberasturi D.J., Paz M.M., Liz-Marzán L.M. (2019). Size-Dependent Transport and Cytotoxicity of Mitomycin-Gold Nanoparticle Conjugates in 2D and 3D Mammalian Cell Models. Bioconjug. Chem..

[112] Francesko A., Petkova P., Tzanov T. (2018). Hydrogel Dressings for Advanced Wound Management. Curr. Med. Chem..

[113] Alven S., Aderibigbe B.A. (2020). Chitosan and Cellulose-Based Hydrogels for Wound Management. Int. J. Mol. Sci..

[114] Zhao X., Wu H., Guo B., Dong R., Qiu Y., Ma P.X. (2017). Antibacterial anti-oxidant electroactive injectable hydrogel as self-healing wound dressing with hemostasis and adhesiveness for cutaneous wound healing. Biomaterials.

[115] Dhaliwal K., Lopez N. (2018). Hydrogel dressings and their application in burn wound care. Br. J. Community Nurs..

[116] Zhang J., Zhu Y., Zhang Y., Lin W., Ke J., Liu J., Zhang L., Liu J. (2021). A balanced charged hydrogel with anti-biofouling and antioxidant properties for treatment of irradiation-induced skin injury. Mater. Sci. Eng. C Mater. Biol. Appl..

[117] Tavakoli S., Klar A.S. (2020). Advanced Hydrogels as Wound Dressings. Biomolecules.

[118] Kharaziha M., Baidya A., Annabi N. (2021). Rational Design of Immunomodulatory Hydrogels for Chronic Wound Healing. Adv. Mater..

[119] Niladri R., Nabanita S., Petr H., Petr S. (2010). Permeability and biocompatibility of novel medicated hydrogel wound dressings. Soft Mater..

[120] Reimer K., Fleischer W., Brögmann B., Schreier H., Burkhard P., Lanzendörfer A., Gümbel H., Hoekstra H., Behrens-Baumann W. (1997). Povidone-iodine liposomes—An overview. Dermatology.

[121] Xu H.L., Chen P.P., ZhuGe D.L., Zhu Q.Y., Jin B.H., Shen B.X., Xiao J., Zhao Y.Z. (2017). Liposomes with Silk Fibroin Hydrogel Core to Stabilize bFGF and Promote the Wound Healing of Mice with Deep Second-Degree Scald. Adv. Healthc. Mater..

[122] Reimer K., Vogt P.M., Broegmann B., Hauser J., Rossbach O., Kramer A., Rudolph P., Bosse B., Schreier H., Fleischer W. (2000). An innovative topical drug formulation for wound healing and infection treatment: In vitro and in vivo investigations of a povidone-iodine liposome hydrogel. Dermatology.

[123] Sağıroğlu A.A., Çelik B., Güler E.M., Koçyiğit A., Özer Ö. (2021). Evaluation of wound healing potential of new composite liposomal films containing coenzyme Q10 and d-panthenyl triacetate as combinational treatment. Pharm. Dev. Technol..

[124] Santos A.C., Rodrigues D., Sequeira J.A.D., Pereira I., Simões A., Costa D., Peixoto D., Costa G., Veiga F. (2019). Nanotechnological breakthroughs in the development of topical phytocompounds-based formulations. Int. J. Pharm..

[125] Shakeel F., Alam P., Anwer M.K., Alanazi S.A., Alsarra I.A., Alqarni M.H. (2019). Wound healing evaluation of self-nanoemulsifying drug delivery system containing Piper cubeba essential oil. 3 Biotech.

[126] Koshak A.E., Algandaby M.M., Mujallid M.I., Abdel-Naim A.B., Alhakamy N.A., Fahmy U.A., Alfarsi A., Badr-Eldin S.M., Neamatallah T., Nasrullah M.Z. (2021). Wound Healing Activity of Opuntia ficus-indica Fixed Oil Formulated in a Self-Nanoemulsifying Formulation. Int. J. Nanomed..

[127] Ponto T., Latter G., Luna G., Leite-Silva V.R., Wright A., Benson H.A.E. (2021). Novel Self-Nano-Emulsifying Drug Delivery Systems Containing Astaxanthin for Topical Skin Delivery. Pharmaceutics.

[128] Khan M., Nadhman A., Sehgal S.A., Siraj S., Yasinzai M.M. (2018). Formulation and characterization of a Self-Emulsifying Drug Delivery System (SEDDS) of curcumin for the topical application in cutaneous and mucocutaneous leishmaniasis. Curr. Top. Med. Chem..

[129] Anand S., Pandey P., Begum M.Y., Chidambaram K., Arya D.K., Gupta R.K., Sankhwar R., Jaiswal S., Thakur S., Rajinikanth P.S. (2022). Electrospun biomimetic multifunctional nanofibers loaded with ferulic acid for enhanced antimicrobial and wound-healing activities in STZ-Induced Diabetic Rats. Pharmaceuticals.

[130] Samadian H., Zamiri S., Ehterami A. (2020). Electrospun cellulose acetate/gelatin nanofibrous wound dressing containing berberine for diabetic foot ulcer healing: In vitro and in vivo studies. Sci. Rep..

[131] Almasian A., Najafi F., Eftekhari M., Shams Ardekani M.R., Sharifzadeh M., Khanavi M. (2021). Preparation of Polyurethane/Pluronic F127 Nanofibers Containing Peppermint Extract Loaded Gelatin Nanoparticles for Diabetic Wounds Healing: Characterization, in vitro, and in vivo Studies. Evid. Based Complement. Altern. Med..

[132] Grip J., Engstad R., Skjæveland I., Škalko-Basnet N., Isaksson J., Basnet P., Holsæter A.M. (2018). Beta-glucan-loaded nanofiber dressing improves wound healing in diabetic mice. Eur. J. Pharm. Sci..

[133] Xu X., Wang X., Qin C., Khan A.U.R., Zhang W., Mo X. (2021). Silk fibroin/poly-(L-lactide-co-caprolactone) nanofiber scaffolds loaded with Huangbai Liniment to accelerate diabetic wound healing. Colloids Surf. B Biointerfaces.

[134] Alzarea A.I., Alruwaili N.K., Ahmad M.M., Munir M.U., Butt A.M., Alrowaili Z.A., Shahari M.S.B., Almalki Z.S., Alqahtani S.S., Dolzhenko A.V. (2022). Development and Characterization of Gentamicin-Loaded Arabinoxylan-Sodium Alginate Films as Antibacterial Wound Dressing. Int. J. Mol. Sci..

[135] Lv F., Wang J., Xu P., Han Y., Ma H., Xu H., Chen S., Chang J., Ke Q., Liu M. (2017). A conducive bioceramic/polymer composite biomaterial for diabetic wound healing. Acta Biomater..

[136] Li Y., Zhang Z.Z. (2018). Sustained curcumin release from PLGA microspheres improves bone formation under diabetic conditions by inhibiting the reactive oxygen species production. Drug Des. Dev. Ther..

[137] Elkomy M.H., Eid H.M., Elmowafy M., Shalaby K., Zafar A., Abdelgawad M.A., Rateb M.E., Ali M.R.A., Alsalahat I., Abou-Taleb H.A. (2022). Bilosomes as a promising nanoplatform for oral delivery of an alkaloid nutraceutical: Improved pharmacokinetic profile and snowballed hypoglycemic effect in diabetic rats. Drug Deliv..

[138] Ternullo S., Schulte Werning L.V., Holsæter A.M., Škalko-Basnet N. (2019). Curcumin-in-Deformable Liposomes-in-Chitosan-Hydrogel as a Novel Wound Dressing. Pharmaceutics.

[139] Cui M.D., Pan Z.H., Pan L.Q. (2017). DangguiBuxue Extract-Loaded Liposomes in Thermosensitive Gel Enhance In Vivo Dermal Wound Healing via Activation of the VEGF/PI3K/Akt and TGF-β/SmadsSignaling Pathway. Evid. Based Complement. Altern. Med..

[140] Kalantari K., Mostafavi E., Afifi A.M., Izadiyan Z., Jahangirian H., Rafiee-Moghaddam R., Webster T.J. (2020). Wound dressings functionalized with silver nanoparticles: Promises and pitfalls. Nanoscale..

[141] Shalaby M.A., Anwar M.M., Saeed H. (2022). Nanomaterials for application in wound Healing: Current state-of-the-art and future perspectives. J. Polym. Res..

[142] Souriyan-Reyhani pour H., Khajavi R., Yazdanshenas M.E., Zahedi P., Mirjalili M. (2018). Cellulose acetate/poly(vinyl alcohol) hybrid fibrous mat containing tetracycline hydrochloride and phenytoin sodium: Morphology, drug release, antibacterial, and cell culture studies. J. Bioact. Compat. Polym..

[143] Kong Y., Xu R., Darabi M.A., Zhong W., Luo G., Xing M.M.Q., Wu J. (2016). Fast and safe fabrication of a free-standing chitosan/alginate nanomembrane to promote stem cell delivery and wound healing. Int. J. Nanomed..

[144] Lohmann N., Schirmer L., Atallah P., Wandel E., Ferrer R.A., Werner C., Simon J.C., Franz S., Freudenberg U. (2017). Glycosaminoglycan-based hydrogels capture inflammatory chemokines and rescue defective wound healing in mice. Sci. Transl. Med..

[145] Lu K.J., Wang W., Xu X.L., Jin F.Y., Qi J., Wang X.J., Kang X.Q., Zhu M.L., Huang Q.L., Yu C.H. (2019). A dual deformable liposomal ointment functionalized with retinoic acid and epidermal growth factor for enhanced burn wound healing therapy. Biomater. Sci..

[146] Tao B., Lin C., Deng Y., Yuan Z., Shen X., Chen M., He Y., Peng Z., Hu Y., Cai K. (2019). Copper-nanoparticle-embedded hydrogel for killing bacteria and promoting wound healing with photothermal therapy. J. Mater. Chem. B.

[147] Cao L., Shao G., Ren F., Yang M., Nie Y., Peng Q., Zhang P. (2021). Cerium oxide nanoparticle-loaded polyvinyl alcohol nanogels delivery for wound healing care systems on surgery. Drug Deliv..

[148] Ziv-Polat O., Topaz M., Brosh T., Margel S. (2010). Enhancement of incisional wound healing by thrombin conjugated iron oxide nanoparticles. Biomaterials.

[149] Walker R.M., Gillespie B.M., Thalib L., Higgins N.S., Whitty J.A. (2017). Foam dressings for treating pressure ulcers. Cochrane Database Syst. Rev..

[150] Xue Y., Chen C., Tan R., Zhang J., Fang Q., Jin R., Mi X., Sun D., Xue Y., Wang Y. (2022). Artificial Intelligence-Assisted Bioinformatics, Microneedle, and Diabetic Wound Healing: A "New Deal" of an Old Drug. ACS Appl. Mater. Interfaces.

[151] Yao S., Wang Y., Chi J., Yu Y., Zhao Y., Luo Y., Wang Y. (2022). Porous MOF Microneedle Array Patch with Photothermal Responsive Nitric Oxide Delivery for Wound Healing. Adv. Sci..

[152] Shan Y., Tan B., Zhang M., Xie X., Liao J. (2022). Restorative biodegradable two-layered hybrid microneedles for melanoma photothermal/chemo co-therapy and wound healing. J. Nanobiotechnol..

[153] Sillmon K., Moran C., Shook L., Lawson C., Burfield A.H. (2021). The Use of Prophylactic Foam Dressings for Prevention of Hospital-Acquired Pressure Injuries: A Systematic Review. J. Wound Ostomy Cont. Nurs..

[154] Sierra-Sánchez Á., Kim K.H., Blasco-Morente G., Arias-Santiago S. (2021). Cellular human tissue-engineered skin substitutes investigated for deep and difficult to heal injuries. NPJ Regen. Med..

[155] Jin S., Oh Y.N., Son Y.R., Kwon B., Park J.H., Gang M.J., Kim B.W., Kwon H.J. (2022). Three-Dimensional Skin Tissue Printing with Human Skin Cell Lines and Mouse Skin-Derived Epidermal and Dermal Cells. J Microbiol. Biotechnol..

[156] Tan S.H., Ngo Z.H., Sci D.B., Leavesley D., Liang K. (2022). Recent Advances in the Design of Three-Dimensional and Bioprinted Scaffolds for Full-Thickness Wound Healing. Tissue Eng. Part B Rev..

[157] Varkey M., Visscher D.O., van Zuijlen P.P.M., Atala A., Yoo J.J. (2019). Skin bioprinting: The future of burn wound reconstruction?. Burns. Trauma.

[158] Gupta P., Sheikh A., Abourehab M.A.S., Kesharwani P. (2022). Amelioration of Full-Thickness Wound Using Hesperidin Loaded Dendrimer-Based Hydrogel Bandages. Biosensors.

[159] Vedhanayagam M., Unni Nair B., Sreeram K.J. (2018). Collagen-ZnO Scaffolds for Wound Healing Applications: Role of Dendrimer Functionalization and Nanoparticle Morphology. ACS Appl. Bio. Mater..

[160] Patrulea V., Borchard G., Jordan O. (2020). An Update on Antimicrobial Peptides (AMPs) and Their Delivery Strategies for Wound Infections. Pharmaceutics.

[161] Jiang Y., Zhao W., Xu S., Wei J., Lasaosa F.L., He Y., Mao H., BoleaBailo R.M., Kong D., Gu Z. (2022). Bioinspired design of mannose-decorated globular lysine dendrimers promotes diabetic wound healing by orchestrating appropriate macrophage polarization. Biomaterials.

[162] Wang Y., Sun H. (2021). Polymeric Nanomaterials for Efficient Delivery of Antimicrobial Agents. Pharmaceutics.

[163] Jiang G., Liu S., Yu T., Wu R., Ren Y., van der Mei H.C., Liu J., Busscher H.J. (2021). PAMAM dendrimers with dual-conjugated vancomycin and Ag-nanoparticles do not induce bacterial resistance and kill vancomycin-resistant Staphylococci. Acta Biomater..

[164] Abdel-Sayed P., Kaeppeli A., Siriwardena T., Darbre T., Perron K., Jafari P., Reymond J.L., Pioletti D.P., Applegate L.A. (2016). Anti-Microbial Dendrimers against Multidrug-Resistant P. aeruginosa Enhance the Angiogenic Effect of Biological Burn-wound Bandages. Sci. Rep..

[165] Dongargaonkar A.A., Bowlin G.L., Yang H. (2013). Electrospun blends of gelatin and gelatin-dendrimer conjugates as a wound-dressing and drug-delivery platform. Biomacromolecules.

[166] Zhang Y., Wang B., Meng X., Sun G., Gao C. (2011). Influences of acid-treated multiwalled carbon nanotubes on fibroblasts: Proliferation, adhesion, migration, and wound healing. Ann. Biomed. Eng..

[167] Kittana N., Assali M., Abu-Rass H., Lutz S., Hindawi R., Ghannam L., Zakarneh M., Mousa A. (2018). Enhancement of wound healing by single-wall/multi-wall carbon nanotubes complexed with chitosan. Int. J. Nanomed..

[168] Liu S., Wu G., Chen X., Zhang X., Yu J., Liu M., Zhang Y., Wang P. (2019). Degradation Behavior In Vitro of Carbon Nanotubes (CNTs)/Poly(lactic acid) (PLA) Composite Suture. Polymers.

[169] Liu J., Ismail N.A., Yusoff M., Razali M.H. (2022). Physicochemical Properties and Antibacterial Activity of Gellan Gum Incorporating Zinc Oxide/Carbon Nanotubes Bionanocomposite Film for Wound Healing. Bioinorg. Chem. Appl..

[170] Khalid A., Madni A., Raza B., Islam M.U., Hassan A., Ahmad F., Ali H., Khan T., Wahid F. (2022). Multiwalled carbon nanotubes functionalized bacterial cellulose as an efficient healing material for diabetic wounds. Int. J. Biol. Macromol..

[171] Chen G., Wu Y., Yu D., Li R., Luo W., Ma G., Zhang C. (2019). Isoniazid-loaded chitosan/carbon nanotubes microspheres promote secondary wound healing of bone tuberculosis. J. Biomater. Appl..

[172] Zhao D., Jing H., Li X., Zhao W. (2021). Application of Nano-Composite Technology for Multi-Empty Carbon Nanotubes in Dressing Change Care. J. Nanosci. Nanotechnol..

[173] Yin M., Wu J., Deng M., Wang P., Ji G., Wang M., Zhou C., Blum N.T., Zhang W., Shi H. (2021). Multifunctional Magnesium Organic Framework-Based Microneedle Patch for Accelerating Diabetic Wound Healing. ACS Nano.

[174] Wang Y., Lu H., Guo M., Chu J., Gao B., He B. (2022). Personalized and Programmable Microneedle Dressing for Promoting Wound Healing. Adv. Healthc. Mater..

[175] Chi J., Zhang X., Chen C., Shao C., Zhao Y., Wang Y. (2020). Antibacterial and angiogenic chitosan microneedle array patch for promoting wound healing. Bioact. Mater..

[176] Chi J., Sun L., Cai L., Fan L., Shao C., Shang L., Zhao Y. (2021). Chinese herb microneedle patch for wound healing. Bioact. Mater..

[177] Wang Y., Gao B., He B. (2022). Toward Efficient Wound Management: Bioinspired Microfluidic and Microneedle Patch. Small.

[178] Liu A., Wang Q., Zhao Z., Wu R., Wang M., Li J., Sun K., Sun Z., Lv Z., Xu J. (2021). Nitric Oxide Nanomotor Driving Exosomes-Loaded Microneedles for Achilles Tendinopathy Healing. ACS Nano.

[179] Gao S., Zhang W., Zhai X., Zhao X., Wang J., Weng J., Li J., Chen X. (2023). An antibacterial and proangiogenic double-layer drug-loaded microneedle patch for accelerating diabetic wound healing. Biomater. Sci..

[180] Xu F.W., Lv Y.L., Zhong Y.F., Xue Y.N., Wang Y., Zhang L.Y., Hu X., Tan W.Q. (2021). Beneficial Effects of Green Tea EGCG on Skin Wound Healing: A Comprehensive Review. Molecules.

[181] Barnum L., Samandari M., Schmidt T.A., Tamayol A. (2020). Microneedle arrays for the treatment of chronic wounds. Expert Opin. Drug Deliv..

[182] Faraji Rad Z., Prewett P.D., Davies G.J. (2021). An overview of microneedle applications, materials, and fabrication methods. Beilstein J. Nanotechnol..

